# Logical Entropy: Introduction to Classical and Quantum Logical Information Theory

**DOI:** 10.3390/e20090679

**Published:** 2018-09-06

**Authors:** David Ellerman

**Affiliations:** Department of Philosophy, University of California Riverside, Riverside, CA 92521, USA; david@ellerman.org

**Keywords:** logical entropy, partition logic, qudits of an observable

## Abstract

Logical information theory is the quantitative version of the logic of partitions just as logical probability theory is the quantitative version of the dual Boolean logic of subsets. The resulting notion of information is about distinctions, differences and distinguishability and is formalized using the distinctions (“dits”) of a partition (a pair of points distinguished by the partition). All the definitions of simple, joint, conditional and mutual entropy of Shannon information theory are derived by a uniform transformation from the corresponding definitions at the logical level. The purpose of this paper is to give the direct generalization to quantum logical information theory that similarly focuses on the pairs of eigenstates distinguished by an observable, i.e., qudits of an observable. The fundamental theorem for quantum logical entropy and measurement establishes a direct quantitative connection between the increase in quantum logical entropy due to a projective measurement and the eigenstates (cohered together in the pure superposition state being measured) that are distinguished by the measurement (decohered in the post-measurement mixed state). Both the classical and quantum versions of logical entropy have simple interpretations as “two-draw” probabilities for distinctions. The conclusion is that quantum logical entropy is the simple and natural notion of information for quantum information theory focusing on the distinguishing of quantum states.

## 1. Introduction

The formula for “classical” logical entropy goes back to the early Twentieth Century [1]. It is the derivation of the formula from basic logic that is new and accounts for the name. The ordinary Boolean logic of subsets has a dual logic of partitions [2] since partitions (=equivalence relations = quotient sets) are category-theoretically dual to subsets. Just as the quantitative version of subset logic is the notion of logical finite probability, so the quantitative version of partition logic is logical information theory using the notion of logical entropy [3]. This paper generalizes that “classical” (i.e., non-quantum) logical information theory to the quantum version. The classical logical information theory is briefly developed before turning to the quantum version. Applications of logical entropy have already been developed in several special mathematical settings; see [4] and the references cited therein.

## 2. Duality of Subsets and Partitions

**Figure 1:**
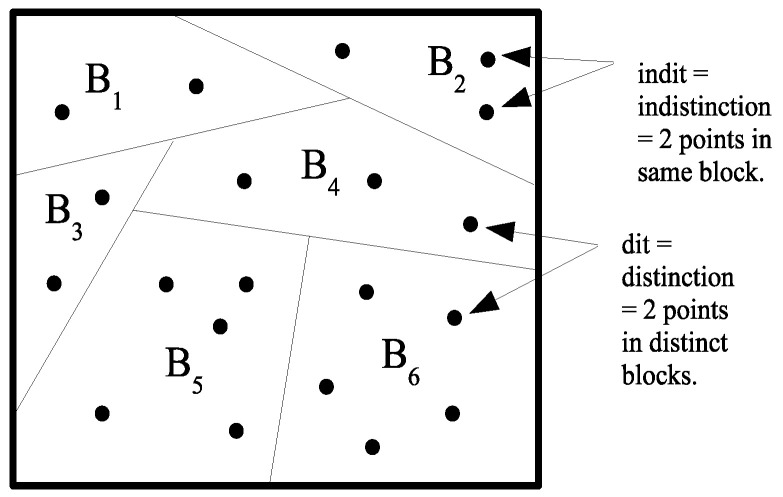
Distinctions and indistinctions of a partition.

The foundations for classical and quantum logical information theory are built on the logic of partitions ([2,5]), which is dual (in the category-theoretic sense) to the usual Boolean logic of subsets. This duality can be most simply illustrated using a set function f:X→Y. The image $f\left(X\right)$ is a *subset* of the codomain *Y* and the inverse-image or coimage $f^{-1}\left(Y\right)$ is a *partition* on the domain *X*, where a *partition* $\pi =\left\{B_{1},\cdots ,B_{I}\right\}$ on a set *U* is a set of subsets or blocks $B_{i}$ that are mutually disjoint and jointly exhaustive ($\cup_{i}B_{i}=U$) In category theory, the duality between subobject-type constructions (e.g., limits) and quotient-object-type constructions (e.g., colimits) is often indicated by adding the prefix “co-” to the latter. Hence, the usual Boolean logic of “images” has the dual logic of “coimages”. However, the duality runs deeper than between subsets and partitions. The dual to the notion of an “element” (an “it”) of a subset is the notion of a “distinction” (a “dit”) of a partition, where $\left(u,u^{\prime }\right)\in U\times U$ is a distinction or dit of π if the two elements are in different blocks. Let $\mathrm{dit}\left(\pi \right)\subseteq U\times U$ be the set of distinctions or *ditset* of π. Similarly an *indistinction* or *indit* of π is a pair $\left(u,u^{\prime }\right)\in U\times U$ in the same block of π. Let $\mathrm{indit}\left(\pi \right)\subseteq U\times U$ be the set of indistinctions or *inditset* of π. Then, $\mathrm{indit}\left(\pi \right)$ is the equivalence relation associated with π, and $\mathrm{dit}\left(\pi \right)=U\times U-\mathrm{indit}\left(\pi \right)$ is the complementary binary relation that might be called *apartition relation* or an *a partness relation*. The notions of a distinction and indistinction of a partition are illustrated in Figure 1.

**Figure 2:**
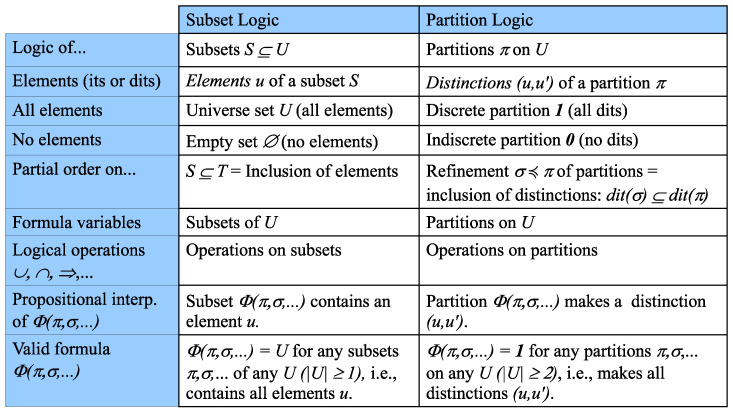
Dual logics: Boolean subset logic of subsets and partition logic.

The relationships between Boolean subset logic and partition logic are summarized in Figure 2, which illustrates the dual relationship between the elements (“its”) of a subset and the distinctions (“dits”) of a partition.

## 3. From the Logic of Partitions to Logical Information Theory

In Gian-Carlo Rota’s Fubini Lectures [6] (and in his lectures at MIT), he remarked in view of duality between partitions and subsets that, quantitatively, the “lattice of partitions plays for information the role that the Boolean algebra of subsets plays for size or probability” ([7] p. 30) or symbolically:

$$\frac{\mathrm{Logical}\;\mathrm{Probability}\;\mathrm{Theory}}{\mathrm{Boolean}\;\mathrm{Logic}\;\mathrm{of}\;\mathrm{Subsets}}=\frac{\mathrm{Logical}\;\mathrm{Information}\;\mathrm{Theory}}{\mathrm{Logic}\;\mathrm{of}\;\mathrm{Partitions}}.$$

Andrei Kolmogorov has suggested that information theory should start with sets, not probabilities.

Information theory must precede probability theory, and not be based on it. By the very essence of this discipline, the foundations of information theory have a finite combinatorial character.

([8] p. 39)

The notion of information-as-distinctions does start with the *set of distinctions*, the *information set*, of a partition $\pi =\left\{B_{1},\cdots ,B_{I}\right\}$ on a finite set *U* where that set of distinctions (dits) is:

$$\mathrm{dit}\left(\pi \right)=\left\{\left(u,u^{\prime }\right):\exists B_{i},B_{i^{\prime }}\in \pi ,B_{i}\neq B_{i^{\prime }},u\in B_{i},u^{\prime }\in B_{i^{\prime }}\right\}.$$

The normalized size of a subset is the logical probability of the event, and the normalized size of the ditset of a partition is, in the sense of measure theory, the measure of the amount of information in a partition. Thus, we define the *logical entropy* of a partition $\pi =\left\{B_{1,}\cdots ,B_{I}\right\}$, denoted $h\left(\pi \right)$, as the size of the ditset $\mathrm{dit}\left(\pi \right)\subseteq U\times U$ normalized by the size of U×U:

$$h\left(\pi \right)=\frac{\left|\mathrm{dit}\left(\pi \right)\right|}{\left|U\times U\right|}=\frac{\left|U\times U\right|-\sum_{i=1}^{I}\left|B_{i}\times B_{i}\right|}{\left|U\times U\right|}=1-\sum_{i=1}^{I}{\left(\frac{\left|B_{i}\right|}{\left|U\right|}\right)}^{2}=1-\sum_{i=1}^{I}\mathrm{Pr}{\left(B_{i}\right)}^{2}.$$

In two independent draws from *U*, the probability of getting a distinction of π is the probability of not getting an indistinction.

Given any probability measure p:U→[0,1] on $U=\left\{u_{1},\cdots ,u_{n}\right\}$, which defines $p_{i}=p\left(u_{i}\right)$ for i=1,⋯,n, the *product measure* $p\times p:U\times U\to \left[0,1\right]$ has for any binary relation R⊆U×U the value of:

$$p\times p\left(R\right)=\sum_{\left(u_{i},u_{j}\right)\in R}p\left(u_{i}\right)p\left(u_{j}\right)=\sum_{\left(u_{i},u_{j}\right)\in R}p_{i}p_{j}.$$

The *logical entropy* of π in general is the product-probability measure of its ditset $\mathrm{dit}\left(\pi \right)\subseteq U\times U$, where $\mathrm{Pr}\left(B\right)=\sum_{u\in B}p\left(u\right)$:

$$h\left(\pi \right)=p\times p\left(\mathrm{dit}\left(\pi \right)\right)=\sum_{\left(u_{i},u_{j}\right)\in \mathrm{dit}\left(\pi \right)}p_{i}p_{j}=1-\sum_{B\in \pi }\mathrm{Pr}{\left(B\right)}^{2}.$$

The standard interpretation of $h\left(\pi \right)$ is the two-draw probability of getting a distinction of the partition π, just as $\mathrm{Pr}\left(S\right)$ is the one-draw probability of getting an element of the subset-event *S*.

## 4. Compound Logical Entropies

The compound notions of logical entropy are also developed in two stages, first as sets and then, given a probability distribution, as two-draw probabilities. After observing the similarity between the formulas holding for the compound Shannon entropies and the Venn diagram formulas that hold for any measure (in the sense of measure theory), the information theorist, Lorne L. Campbell, remarked in 1965 that the similarity:

suggests the possibility that $H\left(\alpha \right)$ and $H\left(\beta \right)$ are measures of sets, that $H\left(\alpha ,\beta \right)$ is the measure of their union, that $I\left(\alpha ,\beta \right)$ is the measure of their intersection, and that $H\left(\alpha |\beta \right)$ is the measure of their difference. The possibility that $I\left(\alpha ,\beta \right)$ is the entropy of the “intersection” of two partitions is particularly interesting. This “intersection,” if it existed, would presumably contain the information common to the partitions α and β.

([9] p. 113)

Yet, there is no such interpretation of the Shannon entropies as measures of sets, but the logical entropies precisely fulfill Campbell’s suggestion (with the “intersection” of two partitions being the intersection of their ditsets). Moreover, there is a uniform requantifying transformation (see the next section) that obtains all the Shannon definitions from the logical definitions and explains how the Shannon entropies can satisfy the Venn diagram formulas (e.g., as a mnemonic) while not being defined by a measure on sets.

Given partitions $\pi =\left\{B_{1},\cdots ,B_{I}\right\}$ and $\sigma =\left\{C_{1},\cdots ,C_{J}\right\}$ on *U*, the *joint information set* is the union of the ditsets, which is also the *ditset for their join*: $\mathrm{dit}\left(\pi \right)\cup \mathrm{dit}\left(\sigma \right)=\mathrm{dit}\left(\pi \vee \sigma \right)\subseteq U\times U$. Given probabilities $p=\left\{p_{1},\cdots ,p_{n}\right\}$ on *U*, the *joint logical entropy* is the product probability measure on the union of ditsets:

$$h\left(\pi ,\sigma \right)=h\left(\pi \vee \sigma \right)=p\times p\left(\mathrm{dit}\left(\pi \right)\cup \mathrm{dit}\left(\sigma \right)\right)=1-\sum_{i,j}\mathrm{Pr}{\left(B_{i}\cap C_{j}\right)}^{2}.$$

The information set for the *conditional logical entropy* $h\left(\pi |\sigma \right)$ is the difference of ditsets, and thus, that logical entropy is:

$$h\left(\pi |\sigma \right)=p\times p\left(\mathrm{dit}\left(\pi \right)-\mathrm{dit}\left(\sigma \right)\right)=h\left(\pi ,\sigma \right)-h\left(\sigma \right).$$

The information set for the *logical mutual information* $m\left(\pi ,\sigma \right)\;$ is the intersection of ditsets, so that logical entropy is:

$$m\left(\pi ,\sigma \right)=p\times p\left(\mathrm{dit}\left(\pi \right)\cap \mathrm{dit}\left(\sigma \right)\right)=h\left(\pi ,\sigma \right)-h\left(\pi |\sigma \right)-h\left(\sigma |\pi \right)=h\left(\pi \right)+h\left(\sigma \right)-h\left(\pi ,\sigma \right).$$

**Figure 3:**
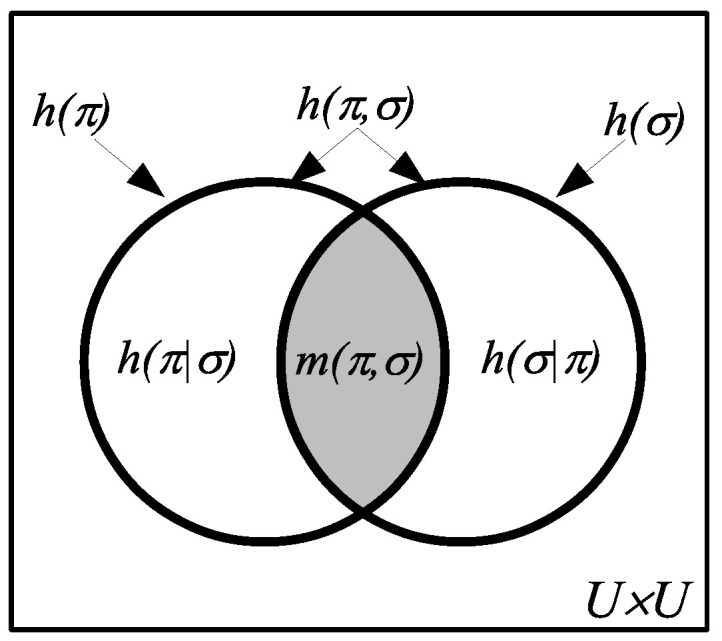
Venn diagram for logical entropies as values of a probability measure p×p on U×U.

Since all the logical entropies are the values of a measure $p\times p:U\times U\to \left[0,1\right]$ on subsets of U×U, they automatically satisfy the usual Venn diagram relationships as in Figure 3.

At the level of information sets (w/o probabilities), we have the *information algebra* $I\left(\pi ,\sigma \right)$, which is the Boolean subalgebra of $℘\left(U\times U\right)$ generated by ditsets and their complements.

## 5. Deriving the Shannon Entropies from the Logical Entropies

Instead of being defined as the values of a measure, the usual notions of simple and compound entropy ‘burst forth fully formed from the forehead’ of Claude Shannon [10] already satisfying the standard Venn diagram relationships (one author surmised that “Shannon carefully contrived for this ‘accident’ to occur” ([11] p. 153)). Since the Shannon entropies are not the values of a measure, many authors have pointed out that these Venn diagram relations for the Shannon entropies can only be taken as “analogies” or “mnemonics” ([9,12]). Logical information theory explains this situation since all the Shannon definitions of simple, joint, conditional and mutual information can be obtained by a uniform requantifying transformation from the corresponding logical definitions, and the transformation preserves the Venn diagram relationships.

This transformation is possible since the logical and Shannon notions of entropy can be seen as two different ways to quantify distinctions; and thus, *both* theories are based on the foundational idea of *information-as-distinctions*.

Consider the canonical case of *n* equiprobable elements, $p_{i}=\frac{1}{n}$. The logical entropy of 1 = $\left\{B_{1},\cdots ,B_{n}\right\}$ where $B_{i}=\left\{u_{i}\right\}$ with $p=\left\{\frac{1}{n},\cdots ,\frac{1}{n}\right\}$ is:

$$\frac{\left|U\times U-\Delta \right|}{\left|U\times U\right|}=\frac{n^{2}-n}{n^{2}}=1-\frac{1}{n}=1-\mathrm{Pr}\left(B_{i}\right).$$

The normalized number of distinctions or ‘dit-count’ of the discrete partition 1 is $1-\frac{1}{n}=1-\mathrm{Pr}\left(B_{i}\right)$. The general case of logical entropy for any $\pi =\left\{B_{1},\cdots ,B_{I}\right\}$ is the average of the dit-counts $1-\mathrm{Pr}\left(B_{i}\right)$ for the canonical cases:

$$h\left(\pi \right)=\sum_{i}\mathrm{Pr}\left(B_{i}\right)\left(1-\mathrm{Pr}\left(B_{i}\right)\right).$$

In the canonical case of $2^{n}$ equiprobable elements, the minimum number of binary partitions (“yes-or-no questions” or “bits”) whose join is the discrete partition $1=\left\{B_{1},\cdots ,B_{2^{n}}\right\}$ with $\mathrm{Pr}\left(B_{i}\right)=\frac{1}{2^{n}}$, i.e., that it takes to uniquely encode each distinct element, is *n*, so the Shannon–Hartley entropy [13] is the canonical bit-count:

$$n=\log_{2}\left(2^{n}\right)=\log_{2}\left(\frac{1}{1/2^{n}}\right)=\log_{2}\left(\frac{1}{\mathrm{Pr}\left(B_{i}\right)}\right).$$

The general case of Shannon entropy is the average of these canonical bit-counts $\log_{2}\left(\frac{1}{\mathrm{Pr}\left(B_{i}\right)}\right)$:

$$H\left(\pi \right)=\sum_{i}\mathrm{Pr}\left(B_{i}\right)\log_{2}\left(\frac{1}{\mathrm{Pr}\left(B_{i}\right)}\right).$$

**Figure 4:**
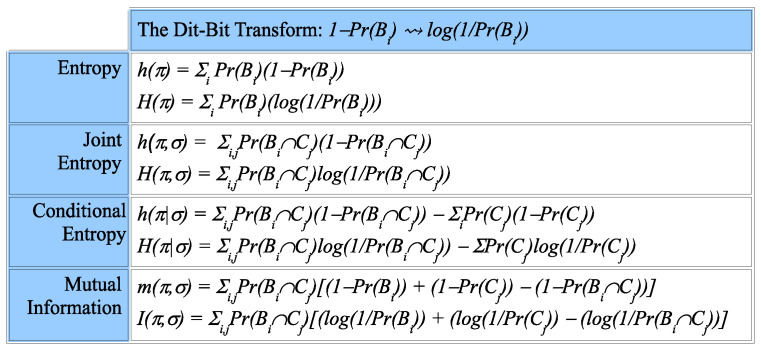
Summary of the dit-bit transform.

The *dit-bit transform* essentially replaces the canonical dit-counts by the canonical bit-counts. First, express any logical entropy concept (simple, joint, conditional or mutual) as an average of canonical dit-counts $1-\mathrm{Pr}\left(B_{i}\right)$, and then, substitute the canonical bit-count $\log\left(\frac{1}{\mathrm{Pr}\left(B_{i}\right)}\right)$ to obtain the corresponding formula as defined by Shannon. Figure 4 gives examples of the dit-bit transform.

For instance,

$$h\left(\pi |\sigma \right)=h\left(\pi ,\sigma \right)-h\left(\sigma \right)=\sum_{i,j}\mathrm{Pr}\left(B_{i}\cap C_{j}\right)\left[1-\mathrm{Pr}\left(B_{i}\cap C_{j}\right)\right]-\sum_{j}\mathrm{Pr}\left(C_{j}\right)\left[1-\mathrm{Pr}\left(C_{j}\right)\right]$$

is the expression for $h\left(\pi |\sigma \right)$ as an average over $1-\mathrm{Pr}\left(B_{i}\cap C_{j}\right)$ and $1-\mathrm{Pr}\left(C_{j}\right)$, so applying the dit-bit transform gives:

$$\sum_{i,j}\mathrm{Pr}\left(B_{i}\cap C_{j}\right)\log\left(1/\mathrm{Pr}\left(B_{i}\cap C_{j}\right)\right)-\sum_{j}\mathrm{Pr}\left(C_{j}\right)\log\left(1/\mathrm{Pr}\left(C_{j}\right)\right)=H\left(\pi ,\sigma \right)-H\left(\sigma \right)=H\left(\pi |\sigma \right).$$

**Figure 5:**
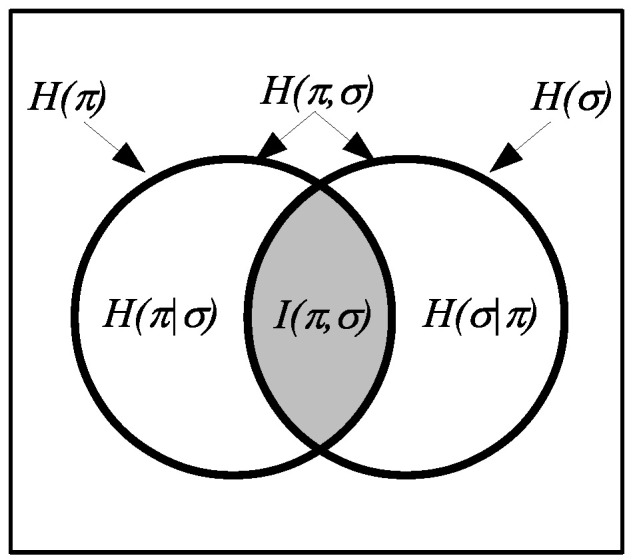
Venn diagram mnemonic for Shannon entropies.

The dit-bit transform is linear in the sense of preserving plus and minus, so the Venn diagram formulas, e.g., $h\left(\pi ,\sigma \right)=h\left(\sigma \right)+h\left(\pi |\sigma \right)$, which are automatically satisfied by logical entropy since it is a measure, carry over to Shannon entropy, e.g., $H\left(\pi ,\sigma \right)=H\left(\sigma \right)+H\left(\pi |\sigma \right)$ as in Figure 5, in spite of it not being a measure (in the sense of measure theory):

## 6. Logical Entropy via Density Matrices

The transition to quantum logical entropy is facilitated by reformulating the classical logical theory in terms of density matrices. Let $U=\left\{u_{1},\cdots ,u_{n}\right\}$ be the sample space with the point probabilities $p=\left(p_{1},\cdots ,p_{n}\right)$. An event S⊆U has the probability $\mathrm{Pr}\left(S\right)=\sum_{u_{j}\in S}p_{j}$.

For any event *S* with $\mathrm{Pr}\left(S\right)>0$, let:

$$|S\rangle =\frac{1}{\sqrt{\mathrm{Pr}\left(S\right)}}{\left(\chi_{S}\left(u_{1}\right)\sqrt{p_{1}},\cdots ,\chi_{S}\left(u_{n}\right)\sqrt{p_{n}}\right)}^{t}$$

(the superscript *t* indicates transpose) which is a normalized column vector in $R^{n}$ where $\chi_{S}:U\to \left\{0,1\right\}$ is the characteristic function for *S*, and let $\langle S|$ be the corresponding row vector. Since $|S\rangle$ is normalized, $\langle S|S\rangle =1$. Then, the *density matrix* representing the event *S* is the n×n symmetric real matrix:

$$\rho \left(S\right)=|S\rangle \langle S|\;\mathrm{so}\;\mathrm{that}\;{\left(\rho \left(S\right)\right)}_{j,k}=\left\{\begin{array}{c} \frac{1}{\mathrm{Pr}\left(S\right)}\sqrt{p_{j}p_{k}}\;\mathrm{for}\;u_{j},u_{k}\in S\\ 0\;\mathrm{otherwise} \end{array}\right..$$

Then, $\rho {\left(S\right)}^{2}=|S\rangle \langle S|S\rangle \langle S|=\rho \left(S\right)$, so borrowing language from quantum mechanics, $\rho \left(S\right)$ is said to be a *pure state* density matrix.

Given any partition $\pi =\left\{B_{1},\cdots ,B_{I}\right\}$ on *U*, its density matrix is the average of the block density matrices:

$$\rho \left(\pi \right)=\sum_{i}\mathrm{Pr}\left(B_{i}\right)\rho \left(B_{i}\right).$$

Then, $\rho \left(\pi \right)$ represents the *mixed state*, experiment or lottery where the event $B_{i}$ occurs with probability $\mathrm{Pr}\left(B_{i}\right)$. A little calculation connects the logical entropy $h\left(\pi \right)$ of a partition with the density matrix treatment:

$$h\left(\pi \right)=1-\sum_{i=1}^{I}\mathrm{Pr}{\left(B_{i}\right)}^{2}=1-\mathrm{tr}\left[\rho {\left(\pi \right)}^{2}\right]=h\left(\rho \left(\pi \right)\right)$$

where $\rho {\left(\pi \right)}^{2}$ is substituted for $\mathrm{Pr}{\left(B_{i}\right)}^{2}$ and the trace is substituted for the summation.

For the throw of a fair die, $U=\left\{u_{1},u_{3},u_{5},u_{2},u_{4},u_{6}\right\}$ (note the odd faces ordered before the even faces in the matrix rows and columns) where $u_{j}$ represents the number *j* coming up, the density matrix $\rho \left(0\right)$ is the “pure state” 6×6 matrix with each entry being $\frac{1}{6}$.

$$\rho \left(0\right)=\left[\begin{array}{cccccc} 1/6 & 1/6 & 1/6 & 1/6 & 1/6 & 1/6\\ 1/6 & 1/6 & 1/6 & 1/6 & 1/6 & 1/6\\ 1/6 & 1/6 & 1/6 & 1/6 & 1/6 & 1/6\\ 1/6 & 1/6 & 1/6 & 1/6 & 1/6 & 1/6\\ 1/6 & 1/6 & 1/6 & 1/6 & 1/6 & 1/6\\ 1/6 & 1/6 & 1/6 & 1/6 & 1/6 & 1/6 \end{array}\right]\begin{array}{c} u_{1}\\ u_{3}\\ u_{5}\\ u_{2}\\ u_{4}\\ u_{6} \end{array}.$$

The nonzero off-diagonal entries represent indistinctions or indits of the partition 0 or, in quantum terms, “coherences” where all 6 “eigenstates” cohere together in a pure “superposition” state. All pure states have a logical entropy of zero, i.e., $h\left(0\right)=0$ (i.e., no dits) since $\mathrm{tr}\left[\rho \right]=1$ for any density matrix, so if $\rho {\left(0\right)}^{2}=\rho \left(0\right)$, then $\mathrm{tr}\left[\rho {\left(0\right)}^{2}\right]=\mathrm{tr}\left[\rho \left(0\right)\right]=1$ and $h\left(0\right)=1-\mathrm{tr}\left[\rho {\left(0\right)}^{2}\right]=0$.

The logical operation of classifying undistinguished entities (like the six faces of the die before a throw to determine a face up) by a numerical attribute makes distinctions between the entities with different numerical values of the attribute. Classification (also called sorting, fibering or partitioning ([14] Section 6.1)) is the classical operation corresponding to the quantum operation of “measurement” of a superposition state by an observable to obtain a mixed state.

Now classify or “measure” the die-faces by the parity-of-the-face-up (odd or even) partition (observable) $\pi =\left\{B_{odd},B_{even}\right\}=\left\{\left\{u_{1},u_{3},u_{5}\right\},\left\{u_{2},u_{4},u_{6}\right\}\right\}$. Mathematically, this is done by the Lüders mixture operation ([15] p. 279), i.e., pre- and post-multiplying the density matrix $\rho \left(0\right)$ by $P_{odd}$ and by $P_{even}$, the projection matrices to the odd or even components, and summing the results:

$$P_{odd}\rho \left(0\right)P_{odd}+P_{even}\rho \left(0\right)P_{even}\;=\left[\begin{array}{cccccc} 1/6 & 1/6 & 1/6 & 0 & 0 & 0\\ 1/6 & 1/6 & 1/6 & 0 & 0 & 0\\ 1/6 & 1/6 & 1/6 & 0 & 0 & 0\\ 0 & 0 & 0 & 0 & 0 & 0\\ 0 & 0 & 0 & 0 & 0 & 0\\ 0 & 0 & 0 & 0 & 0 & 0 \end{array}\right]+\left[\begin{array}{cccccc} 0 & 0 & 0 & 0 & 0 & 0\\ 0 & 0 & 0 & 0 & 0 & 0\\ 0 & 0 & 0 & 0 & 0 & 0\\ 0 & 0 & 0 & 1/6 & 1/6 & 1/6\\ 0 & 0 & 0 & 1/6 & 1/6 & 1/6\\ 0 & 0 & 0 & 1/6 & 1/6 & 1/6 \end{array}\right]\;=\left[\begin{array}{cccccc} 1/6 & 1/6 & 1/6 & 0 & 0 & 0\\ 1/6 & 1/6 & 1/6 & 0 & 0 & 0\\ 1/6 & 1/6 & 1/6 & 0 & 0 & 0\\ 0 & 0 & 0 & 1/6 & 1/6 & 1/6\\ 0 & 0 & 0 & 1/6 & 1/6 & 1/6\\ 0 & 0 & 0 & 1/6 & 1/6 & 1/6 \end{array}\right]\;=\frac{1}{2}\rho \left(B_{odd}\right)+\frac{1}{2}\rho \left(B_{even}\right)=\rho \left(\pi \right).$$

**Theorem** **1** (**Fundamental(classical)**)**.** *The increase in logical entropy, $h\left(\rho \left(\pi \right)\right)-h\left(\rho \left(0\right)\right)$, due to a Lüders mixture operation is the sum of amplitudes squared of the non-zero off-diagonal entries of the beginning density matrix that are zeroed in the final density matrix.*

**Proof.**  Since for any density matrix ρ, $\mathrm{tr}\left[\rho^{2}\right]=\sum_{i,j}{\left|\rho_{ij}\right|}^{2}$ ([16] p. 77), we have: $h\left(\rho \left(\pi \right)\right)-h\left(\rho \left(0\right)\right)=\left(1-\mathrm{tr}\left[\rho {\left(\pi \right)}^{2}\right]\right)-\left(1-\mathrm{tr}\left[\rho {\left(0\right)}^{2}\right]\right)=\mathrm{tr}\left[\rho {\left(0\right)}^{2}\right]-\mathrm{tr}\left[\rho {\left(\pi \right)}^{2}\right]=\sum_{i,j}\left({\left|\rho_{ij}\left(0\right)\right|}^{2}-{\left|\rho_{ij}\left(\pi \right)\right|}^{2}\right)$, if $\left(u_{i},u_{i^{\prime }}\right)\in \mathrm{dit}\left(\pi \right)$, then and only then are the off-diagonal terms corresponding to $u_{i}$ and $u_{i^{\prime }}$ zeroed by the Lüders operation. ☐

The classical fundamental theorem connects the concept of information-as-distinctions to the process of “measurement” or classification, which uses some attribute (like parity in the example) or “observable” to make distinctions.

In the comparison with the matrix $\rho \left(0\right)$ of all entries $\frac{1}{6}$, the entries that got zeroed in the Lüders operation $\rho \left(0\right)⟶\rho \left(\pi \right)$ correspond to the distinctions created in the transition 0 = $\left\{\left\{u_{1},\cdots ,u_{6}\right\}\right\}⟶\pi =\left\{\left\{u_{1},u_{3},u_{5}\right\},\left\{u_{2},u_{4},u_{6}\right\}\right\}$, i.e., the odd-numbered faces were distinguished from the even-numbered faces by the parity attribute. The increase in logical entropy = sum of the squares of the off-diagonal elements that were zeroed = $h\left(\pi \right)-h\left(0\right)=2\times 9\times {\left(\frac{1}{6}\right)}^{2}=\frac{18}{36}=\frac{1}{2}$. The usual calculations of the two logical entropies are: $h\left(\pi \right)=2\times {\left(\frac{1}{2}\right)}^{2}=\frac{1}{2}$ and $h\left(0\right)=1-1=0$.

Since, in quantum mechanics, a projective measurement’s effect on a density matrix *is* the Lüders mixture operation, that means that the effects of the measurement are the above-described “making distinctions” by decohering or zeroing certain coherence terms in the density matrix, and the sum of the absolute squares of the coherences that were decohered is the increase in the logical entropy.

## 7. Quantum Logical Information Theory: Commuting Observables

The idea of information-as-distinctions carries over to quantum mechanics.

[Information] is the notion of distinguishability abstracted away from what we are distinguishing, or from the carrier of information. …And we ought to develop a theory of information which generalizes the theory of distinguishability to include these quantum properties…

([17] p. 155)

Let F:V→V be a self-adjoint operator (observable) on a *n*-dimensional Hilbert space *V* with the real eigenvalues $\phi_{1},\cdots ,\phi_{I}$, and let $U=\left\{u_{1},\cdots ,u_{n}\right\}$ be an orthonormal (ON) basis of eigenvectors of *F*. The quantum version of a dit, a qudit, is a pair of states definitely distinguishable by *some* observable. Any nondegenerate self-adjoint operator such as $\sum_{k=1}^{n}kP_{\left[u_{k}\right]}$, where $P_{\left[u_{k}\right]}$ is the projection to the one-dimensional subspace generated by $u_{k}$, will distinguish all the vectors in the orthonormal basis *U*, which is analogous classically to a pair $\left(u,u^{\prime }\right)$ of distinct elements of *U* that are distinguishable by some partition (i.e., 1). In general, a *qudit* is *relativized to an observable*, just as classically a distinction is a distinction of a *partition*. Then, there is a set partition $\pi ={\left\{B_{i}\right\}}_{i=1,\cdots ,I}$ on the ON basis *U* so that $B_{i}$ is a basis for the eigenspace of the eigenvalue $\phi_{i}$ and $\left|B_{i}\right|$ is the “multiplicity” (dimension of the eigenspace) of the eigenvalue $\phi_{i}$ for i=1,⋯,I. Note that the real-valued function f:U→R takes each eigenvector in $u_{j}\in B_{i}\subseteq U$ to its eigenvalue $\phi_{i}$ so that $f^{-1}\left(\phi_{i}\right)=B_{i}$ contains all the information in the self-adjoint operator F:V→V since *F* can be reconstructed by defining it on the basis *U* as $Fu_{j}=f\left(u_{j}\right)u_{j}$.

The generalization of “classical” logical entropy to quantum logical entropy is straightforward using the usual ways that set-concepts generalize to vector-space concepts: subsets → subspaces, set partitions → direct-sum decompositions of subspaces (hence the “classical” logic of partitions on a set will generalize to the quantum logic of direct-sum decompositions [18] that is the dual to the usual quantum logic of subspaces), Cartesian products of sets → tensor products of vector spaces and ordered pairs $\left(u_{k},u_{k^{\prime }}\right)\in U\times U\to$ basis elements $u_{k}⊗u_{k^{\prime }}\in V⊗V$. The eigenvalue function f:U→R determines a partition ${\left\{f^{-1}\left(\phi_{i}\right)\right\}}_{i\in I}$ on *U*, and the blocks in that partition generate the eigenspaces of *F*, which form a direct-sum decomposition of *V*.

Classically, a *dit of the partition* ${\left\{f^{-1}\left(\phi_{i}\right)\right\}}_{i\in I}$ on *U* is a pair $\left(u_{k},u_{k^{\prime }}\right)$ of points in distinct blocks of the partition, i.e., $f\left(u_{k}\right)\neq f\left(u_{k^{\prime }}\right)$. Hence, a *qudit of* *F* is a pair $\left(u_{k},u_{k^{\prime }}\right)$ (interpreted as $u_{k}⊗u_{k^{\prime }}$ in the context of V⊗V) of vectors in the eigenbasis definitely distinguishable by *F*, i.e., $f\left(u_{k}\right)\neq f\left(u_{k^{\prime }}\right)$, distinct *F*-eigenvalues. Let G:V→V be another self-adjoint operator on *V*, which commutes with *F* so that we may then assume that *U* is an orthonormal basis of simultaneous eigenvectors of *F* and *G*. Let ${\left\{\gamma_{j}\right\}}_{j\in J}$ be the set of eigenvalues of *G*, and let g:U→R be the eigenvalue function so a pair $\left(u_{k},u_{k^{\prime }}\right)$ is a *qudit ofG* if $g\left(u_{k}\right)\neq g\left(u_{k^{\prime }}\right)$, i.e., if the two eigenvectors have distinct eigenvalues of *G*.

**Figure 6:**
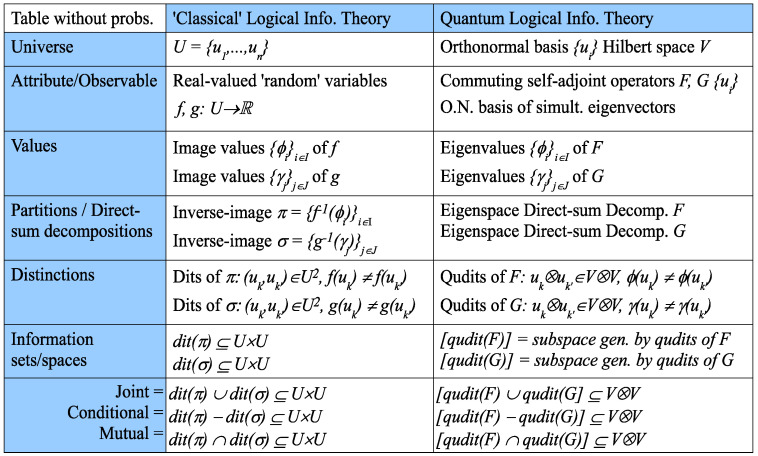
The parallel development of classical and quantum logical information prior to probabilities.

As in classical logical information theory, information is represented by certain subsets (or, in the quantum case, subspaces) prior to the introduction of any probabilities. Since the transition from classical to quantum logical information theory is straightforward, it will be presented in table form in Figure 6 (which does not involve any probabilities), where the qudits $\left(u_{k},u_{k^{\prime }}\right)$ are interpreted as $u_{k}⊗u_{k^{\prime }}$.

The *information subspace* associated with *F* is the subspace $\left[qudit\left(F\right)\right]\subseteq V⊗V$ generated by the qudits $u_{k}⊗u_{k^{\prime }}$ of *F*. If F=λI is a scalar multiple of the identity *I*, then it has no qudits, so its information space $\left[qudit\left(\lambda I\right)\right]$ is the zero subspace. It is an easy implication of the common dits theorem of classical logical information theory (([19] (Proposition 1)) or ([5] (Theorem 1.4))) that any two nonzero information spaces $\left[qudit\left(F\right)\right]$ and $\left[qudit\left(G\right)\right]$ have a nonzero intersection, i.e., have a nonzero mutual information space. That is, there are always two eigenvectors $u_{k}$ and $u_{k^{\prime }}$ that have different eigenvalues both by *F* and by *G*.

**Figure 7:**
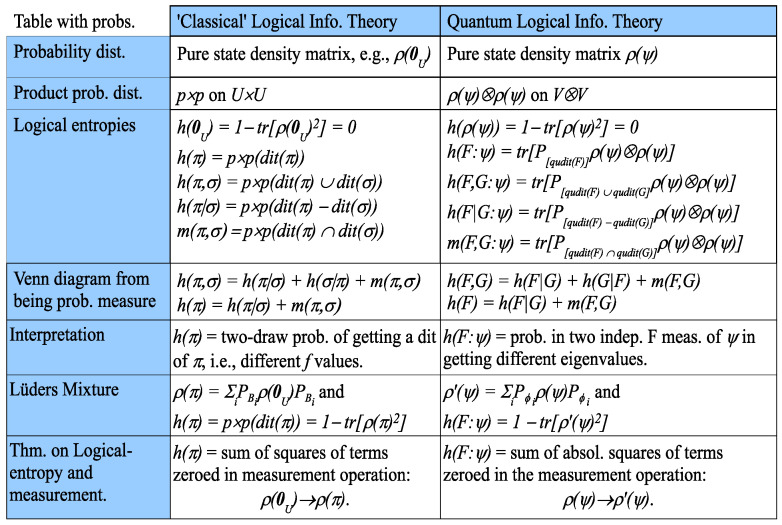
The development of classical and quantum logical entropies for commuting *F* and *G*.

In a measurement, the observables do not provide the point probabilities; they come from the pure (normalized) state ψ being measured. Let $|\psi \rangle =\sum_{j=1}^{n}\langle u_{j}|\psi \rangle |u_{j}\rangle =\sum_{j=1}^{n}\alpha_{j}|u_{j}\rangle$ be the resolution of $|\psi \rangle$ in terms of the orthonormal basis $U=\left\{u_{1},\cdots ,u_{n}\right\}$ of simultaneous eigenvectors for *F* and *G*. Then, $p_{j}=\alpha_{j}\alpha_{j}^{*}$ ($\alpha_{j}^{*}$ is the complex conjugate of $\alpha_{j}$) for j=1,⋯,n are the point probabilities on *U*, and the pure state density matrix $\rho \left(\psi \right)=|\psi \rangle \langle \psi |$ (where $\langle \psi |={|\psi \rangle }^{†}$ is the conjugate-transpose) has the entries: $\rho_{jk}\left(\psi \right)=\alpha_{j}\alpha_{k}^{*}$, so the diagonal entries $\rho_{jj}\left(\psi \right)=\alpha_{j}\alpha_{j}^{*}=p_{j}$ are the point probabilities. Figure 7 gives the remaining parallel development with the probabilities provided by the pure state ψ where we write $\rho {\left(\psi \right)}^{†}\rho \left(\psi \right)$ as $\rho {\left(\psi \right)}^{2}$.

The formula $h\left(\rho \right)=1-\mathrm{tr}\left[\rho^{2}\right]$ is hardly new. Indeed, $\mathrm{tr}\left[\rho^{2}\right]$ is usually called the *purity* of the density matrix since a state ρ is *pure* if and only if $\mathrm{tr}\left[\rho^{2}\right]=1$, so $h\left(\rho \right)=0$, and otherwise, $\mathrm{tr}\left[\rho^{2}\right]\;<1$, so $h\left(\rho \right)>0$; and the state is said to be *mixed*. Hence, the complement $1-\mathrm{tr}\left[\rho^{2}\right]$ has been called the “mixedness” ([20] p. 5) or “impurity” of the state ρ. The seminal paper of Manfredi and Feix [21] approaches the same formula $1-\mathrm{tr}\left[\rho^{2}\right]$ (which they denote as $S_{2}$) from the viewpoint of Wigner functions, and they present strong arguments for this notion of quantum entropy (thanks to a referee for this important reference to the Manfredi and Feix paper). This notion of quantum entropy is also called by the misnomer “linear entropy” even though it is quadratic in ρ, so we will not continue that usage. See [22] or [23] for references to that literature. The logical entropy is also the quadratic special case of the Tsallis–Havrda–Charvat entropy ([24,25]) and the logical distance special case [19] of C. R. Rao’s quadratic entropy [26].

What is new here is not the formula, but the whole back story of partition logic outlined above, which gives the logical notion of entropy arising out of partition logic as the normalized counting measure on ditsets of partitions; just as logical probability arises out of Boolean subset logic as the normalized counting measure on subsets. The basic idea of information is differences, distinguishability and distinctions ([3,19]), so the logical notion of entropy is the measure of the distinctions or dits of a partition, and the corresponding quantum version is the measure of the qudits of an observable.

## 8. Two Theorems about Quantum Logical Entropy

Classically, a pair of elements $\left(u_{j},u_{k}\right)$ either “cohere” together in the same block of a partition on *U*, i.e., are an indistinction of the partition, or they do not, i.e., they are a distinction of the partition. In the quantum case, the nonzero off-diagonal entries $\alpha_{j}\alpha_{k}^{*}$ in the pure state density matrix $\rho \left(\psi \right)=|\psi \rangle \langle \psi |$ are called quantum “coherences” ([27] p. 303; [15] p. 177) because they give the amplitude of the eigenstates $|u_{j}\rangle$ and $|u_{k}\rangle$ “cohering” together in the coherent superposition state vector $|\psi \rangle =\sum_{j=1}^{n}\langle u_{j}|\psi \rangle |u_{j}\rangle =\sum_{j}\alpha_{j}|u_{j}\rangle$. The coherences are classically modeled by the nonzero off-diagonal entries $\sqrt{p_{j}p_{k}}$ for the indistinctions $\left(u_{j},u_{k}\right)\in B_{i}\times B_{i}$, i.e., coherences ≈ indistinctions.

For an observable *F*, let ϕ:U→R be for *F*-eigenvalue function assigning the eigenvalue $\phi \left(u_{i}\right)=\phi_{i}$ for each $u_{i}$ in the ON basis $U=\left\{u_{1},\cdots ,u_{n}\right\}$ of *F*-eigenvectors. The range of ϕ is the set of *F*-eigenvalues $\left\{\phi_{1},\cdots ,\phi_{I}\right\}$. Let $P_{\phi_{i}}:V\to V$ be the projection matrix in the *U*-basis to the eigenspace of $\phi_{i}$. The projective *F*-measurement of the state ψ transforms the pure state density matrix $\rho \left(\psi \right)$ (represented in the ON basis *U* of *F*-eigenvectors) to yield the Lüders mixture density matrix $\rho^{\prime }\left(\psi \right)=\sum_{i=1}^{I}P_{\phi_{i}}\rho \left(\psi \right)P_{\phi_{i}}$ ([15] p. 279). The off-diagonal elements of $\rho \left(\psi \right)$ that are zeroed in $\rho^{\prime }\left(\psi \right)$ are the coherences (quantum indistinctions or *quindits*) that are turned into “decoherences” (quantum distinctions or qudits of the observable being measured).

For any observable *F* and a pure state ψ, a quantum logical entropy was defined as $h\left(F:\psi \right)=\mathrm{tr}\left[P_{\left[qudit\left(F\right)\right]}\rho \left(\psi \right)⊗\rho \left(\psi \right)\right]$. That definition was the quantum generalization of the “classical” logical entropy defined as $h\left(\pi \right)=p\times p\left(\mathrm{dit}\left(\pi \right)\right)$. When a projective *F*-measurement is performed on ψ, the pure state density matrix $\rho \left(\psi \right)$ is transformed into the mixed state density matrix by the quantum Lüders mixture operation, which then defines the quantum logical entropy $h\left(\rho^{\prime }\left(\psi \right)\right)=1-\mathrm{tr}\left[\rho^{\prime }{\left(\psi \right)}^{2}\right]$. The first test of how the quantum logical entropy notions fit together is showing that these two entropies are the same: $h\left(F:\psi \right)=h\left(\rho^{\prime }\left(\psi \right)\right)$. The proof shows that they are both equal to classical logical entropy of the partition $\pi \left(F:\psi \right)$ defined on the ON basis $U=\left\{u_{1},\cdots ,u_{n}\right\}$ of *F*-eigenvectors by the *F*-eigenvalues with the point probabilities $p_{j}=\alpha_{j}^{*}\alpha_{j}$. That is, the inverse images $B_{i}=\phi^{-1}\left(\phi_{i}\right)$ of the eigenvalue function ϕ:U→R define the eigenvalue partition $\pi \left(F:\psi \right)=\left\{B_{1},\cdots ,B_{I}\right\}$ on the ON basis $U=\left\{u_{1},\cdots ,u_{n}\right\}$ with the point probabilities $p_{j}=\alpha_{j}^{*}\alpha_{j}$ provided by the state $|\psi \rangle$ for j=1,⋯,n. The classical logical entropy of that partition is: $h\left(\pi \left(F:\psi \right)\right)=1-\sum_{i=1}^{I}p{\left(B_{i}\right)}^{2}$ where $p\left(B_{i}\right)=\sum_{u_{j}\in B_{i}}p_{j}$.

We first show that $h\left(F:\psi \right)=\mathrm{tr}\left[P_{\left[qudit\left(F\right)\right]}\rho \left(\psi \right)⊗\rho \left(\psi \right)\right]=h\left(\pi \left(F:\psi \right)\right)$. Now, $qudit\left(F\right)=\left\{u_{j}⊗u_{k}:\phi \left(u_{j}\right)\neq \phi \left(u_{k}\right)\right\}$, and $\left[qudit\left(F\right)\right]$ is the subspace of V⊗V generated by it. The n×n pure state density matrix $\rho \left(\psi \right)$ has the entries $\rho_{jk}\left(\psi \right)=\alpha_{j}\alpha_{k}^{*}$, and $\rho \left(\psi \right)⊗\rho \left(\psi \right)$ is an $n^{2}\times n^{2}$ matrix. The projection matrix $P_{\left[qudit\left(F\right)\right]}$ is an $n^{2}\times n^{2}$ diagonal matrix with the diagonal entries, indexed by j,k=1,⋯,n: ${\left[P_{\left[qudit\left(F\right)\right]}\right]}_{jjkk}=1$ if $\phi \left(u_{j}\right)\neq \phi \left(u_{k}\right)$ and zero otherwise. Thus, in the product $P_{\left[qudit(F)\right]}\rho \left(\psi \right)⊗\rho \left(\psi \right)$, the nonzero diagonal elements are the $p_{j}p_{k}$ where $\phi \left(u_{j}\right)\neq \phi \left(u_{k}\right)$, and so, the trace is $\sum_{j.k=1}^{n}\left\{p_{j}p_{k}:\phi \left(u_{j}\right)\neq \phi \left(u_{k}\right)\right\}$, which by definition, is $h\left(F:\psi \right)$. Since $\sum_{j=1}^{n}p_{j}=\sum_{i=1}^{I}p\left(B_{i}\right)=1$, ${\left(\sum_{i=1}^{I}p\left(B_{i}\right)\right)}^{2}=1=\sum_{i=1}^{I}p{\left(B_{i}\right)}^{2}+\sum_{i\neq i^{\prime }}p\left(B_{i}\right)p\left(B_{i^{\prime }}\right)$. By grouping the $p_{j}p_{k}$ in the trace according to the blocks of $\pi \left(F:\psi \right)$, we have:

$$h\left(F:\psi \right)=\mathrm{tr}\left[P_{\left[qudit(F)\right]}\rho \left(\psi \right)⊗\rho \left(\psi \right)\right]=\sum_{j.k=1}^{n}\left\{p_{j}p_{k}:\phi \left(u_{j}\right)\neq \phi \left(u_{k}\right)\right\}\;=\sum_{i\neq i^{\prime }}\sum \left\{p_{j}p_{k}:u_{j}\in B_{i},u_{k}\in B_{i^{\prime }}\right\}=\sum_{i\neq i^{\prime }}p\left(B_{i}\right)p\left(B_{i^{\prime }}\right)\;=1-\sum_{i=1}^{I}p{\left(B_{i}\right)}^{2}=h\left(\pi \left(F:\psi \right)\right)$$.

To show that $h\left(\rho^{\prime }\left(\psi \right)\right)=1-\mathrm{tr}\left[\rho^{\prime }{\left(\psi \right)}^{2}\right]=h\left(\pi \left(F:\psi \right)\right)$ for $\rho^{\prime }\left(\psi \right)=\sum_{i=1}^{I}P_{\phi_{i}}\rho \left(\psi \right)P_{\phi_{i}}$, we need to compute $\mathrm{tr}\left[\rho^{\prime }{\left(\psi \right)}^{2}\right]$. An off-diagonal element in $\rho_{jk}\left(\psi \right)=\alpha_{j}\alpha_{k}^{*}$ of $\rho \left(\psi \right)$ survives (i.e., is not zeroed and has the same value) the Lüders operation if and only if $\phi \left(u_{j}\right)=\phi \left(u_{k}\right)$. Hence, the *j*-th diagonal element of $\rho^{\prime }{\left(\psi \right)}^{2}$ is:

$$\sum_{k=1}^{n}\left\{\alpha_{j}^{*}\alpha_{k}\alpha_{j}\alpha_{k}^{*}:\phi \left(u_{j}\right)=\phi \left(u_{k}\right)\right\}=\sum_{k=1}^{n}\left\{p_{j}p_{k}:\phi \left(u_{j}\right)=\phi \left(u_{k}\right)\right\}=p_{j}p\left(B_{i}\right)$$

where $u_{j}\in B_{i}$. Then, grouping the *j*-th diagonal elements for $u_{j}\in B_{i}$ gives $\sum_{u_{j}\in B_{i}}p_{j}p\left(B_{i}\right)=p{\left(B_{i}\right)}^{2}$. Hence, the whole trace is: $\mathrm{tr}\left[\rho^{\prime }{\left(\psi \right)}^{2}\right]=\sum_{i=1}^{I}p{\left(B_{i}\right)}^{2}$, and thus:

$$h\left(\rho^{\prime }\left(\psi \right)\right)=1-\mathrm{tr}\left[\rho^{\prime }{\left(\psi \right)}^{2}\right]=1-\sum_{i=1}^{I}p{\left(B_{i}\right)}^{2}=h\left(F:\psi \right).$$

This completes the proof of the following theorem.

**Theorem** **2.** *$h\left(F:\psi \right)=h\left(\pi \left(F:\psi \right)\right)=h\left(\rho^{\prime }\left(\psi \right)\right)$.*

Measurement creates distinctions, i.e., turns coherences into “decoherences”, which, classically, is the operation of distinguishing elements by classifying them according to some attribute like classifying the faces of a die by their parity. The fundamental theorem about quantum logical entropy and projective measurement, in the density matrix version, shows how the quantum logical entropy created (starting with $h\left(\rho \left(\psi \right)\right)=0$ for the pure state ψ) by the measurement can be computed directly from the coherences of $\rho \left(\psi \right)$ that are decohered in $\rho^{\prime }\left(\psi \right)$.

**Theorem** **3** (**Fundamental** **(quantum)**)**.** *The increase in quantum logical entropy, $h\left(F:\psi \right)=h\left(\rho^{\prime }\left(\psi \right)\right)$, due to the F-measurement of the pure state ψ, is the sum of the absolute squares of the nonzero off-diagonal terms (coherences) in $\rho \left(\psi \right)$ (represented in an ON basis of F-eigenvectors) that are zeroed (‘decohered’) in the post-measurement Lüders mixture density matrix $\rho^{\prime }\left(\psi \right)=\sum_{i=1}^{I}P_{\phi_{i}}\rho \left(\psi \right)P_{\phi_{i}}$.*

**Proof.**  $h\left(\rho^{\prime }\left(\psi \right)\right)-h\left(\rho \left(\psi \right)\right)=\left(1-\mathrm{tr}\left[\rho^{\prime }{\left(\psi \right)}^{2}\right]\right)-\left(1-\mathrm{tr}\left[\rho {\left(\psi \right)}^{2}\right]\right)=\sum_{jk}\left({\left|\rho_{jk}\left(\psi \right)\right|}^{2}-{\left|\rho_{jk}^{\prime }\left(\psi \right)\right|}^{2}\right)$. If $u_{j}$ and $u_{k}$ are a qudit of *F*, then and only then are the corresponding off-diagonal terms zeroed by the Lüders mixture operation $\sum_{i=1}^{I}P_{\phi_{i}}\rho \left(\psi \right)P_{\phi_{i}}$ to obtain $\rho^{\prime }\left(\psi \right)$ from $\rho \left(\psi \right)$. ☐

Density matrices have long been a standard part of the machinery of quantum mechanics. The fundamental theorem for logical entropy and measurement shows there is a simple, direct and quantitative connection between density matrices and logical entropy. The theorem directly connects the changes in the density matrix due to a measurement (sum of absolute squares of zeroed off-diagonal terms) with the increase in logical entropy due to the *F*-measurement $h\left(F:\psi \right)=\mathrm{tr}\left[P_{\left[qudit(F)\right]}\rho \left(\psi \right)⊗\rho \left(\psi \right)\right]=h\left(\rho^{\prime }\left(\psi \right)\right)$ (where $h\left(\rho \left(\psi \right)\right)=0$ for the pure state ψ).

This direct quantitative connection between state discrimination and quantum logical entropy reinforces the judgment of Boaz Tamir and Eliahu Cohen ([28,29]) that quantum logical entropy is a natural and informative entropy concept for quantum mechanics.

We find this framework of partitions and distinction most suitable (at least conceptually) for describing the problems of quantum state discrimination, quantum cryptography and in general, for discussing quantum channel capacity. In these problems, we are basically interested in a distance measure between such sets of states, and this is exactly the kind of knowledge provided by logical entropy [Reference to [19]].

([28] p. 1)

Moreover, the quantum logical entropy has a simple “two-draw probability” interpretation, i.e., $h\left(F:\psi \right)=h\left(\rho^{\prime }\left(\psi \right)\right)$ is the probability that two independent *F*-measurements of ψ will yield distinct *F*-eigenvalues, i.e., will yield a qudit of *F*. In contrast, the von Neumann entropy has no such simple interpretation, and there seems to be no such intuitive connection between pre- and post-measurement density matrices and von Neumann entropy, although von Neumann entropy also increases in a projective measurement ([30] Theorem 11.9, p. 515).

The development of the quantum logical concepts for two non-commuting operators (see Appendix B) is the straightforward vector space version of the classical logical entropy treatment of partitions on two set *X* and *Y* (see Appendix A).

## 9. Quantum Logical Entropies of Density Operators

The extension of the classical logical entropy $h\left(p\right)=1-\sum_{i=1}^{n}p_{i}^{2}$ of a probability distribution $p=\left(p_{1},\cdots ,p_{n}\right)$ to the quantum case is $h\left(\rho \right)=1-\mathrm{tr}\left[\rho^{2}\right]$ where a density matrix ρ replaces the probability distribution *p* and the trace replaces the summation.

An arbitrary density operator ρ, representing a pure or mixed state on *V*, is also a self-adjoint operator on *V*, so quantum logical entropies can be defined where density operators play the double role of providing the measurement basis (as self-adjoint operators), as well as the state being measured.

Let ρ and τ be two non-commuting density operators on *V*. Let $X={\left\{u_{i}\right\}}_{i=1,\cdots ,n}$ be an orthonormal (ON) basis of ρ eigenvectors, and let ${\left\{\lambda_{i}\right\}}_{i=1,\cdots ,n}$ be the corresponding eigenvalues, which must be non-negative and sum to one, so they can be interpreted as probabilities. Let $Y={\left\{v_{j}\right\}}_{j=1,\cdots ,n}$ be an ON basis of eigenvectors for τ, and let ${\left\{\mu_{j}\right\}}_{j=1,\cdots ,n}$ be the corresponding eigenvalues, which are also non-negative and sum to one.

Each density operator plays a double role. For instance, ρ acts as the observable to supply the measurement basis of ${\left\{u_{i}\right\}}_{i}$ and the eigenvalues ${\left\{\lambda_{i}\right\}}_{i}$, as well as being the state to be measured supplying the probabilities ${\left\{\lambda_{i}\right\}}_{i}$ for the measurement outcomes. In this section, we define quantum logical entropies using the discrete partition $1_{X}$ on the set of “index” states $X={\left\{u_{i}\right\}}_{i}$ and similarly for the discrete partition $1_{Y}$ on $Y={\left\{v_{j}\right\}}_{j}$, the ON basis of eigenvectors for τ.

The qudit sets of $\left(V⊗V\right)⊗\left(V⊗V\right)$ are then defined according to the identity and difference on the index sets and independent of the eigenvalue-probabilities, e.g., $qudit\left(1_{X}\right)=\left\{\left(u_{i}⊗v_{j}\right)⊗\left(u_{i^{\prime }}⊗v_{j^{\prime }}\right):i\neq i^{\prime }\right\}$. The, n the qudit subspaces are the subspaces of ${\left(V⊗V\right)}^{2}$ generated by the qudit sets of generators:

- $\left[qudit\left(1_{X}\right)\right]=\left[\left(u_{i}⊗v_{j}\right)⊗\left(u_{i^{\prime }}⊗v_{j^{\prime }}\right):i\neq i^{\prime }\right]$;
- $\left[qudit\left(1_{Y}\right)\right]=\left[\left(u_{i}⊗v_{j}\right)⊗\left(u_{i^{\prime }}⊗v_{j^{\prime }}\right):j\neq j^{\prime }\right]$;
- $\left[qudit\left(1_{X},1_{Y}\right)\right]=\left[qudit\left(1_{X}\right)\cup qudit\left(1_{Y}\right)\right]=\left[\left(u_{i}⊗v_{j}\right)⊗\left(u_{i^{\prime }}⊗v_{j^{\prime }}\right):i\neq i^{\prime }\;\mathrm{or}\;j\neq j^{\prime }\right]$;
- $\left[qudit\left(1_{X}|1_{Y}\right)\right]=\left[qudit\left(1_{X}\right)-qudit\left(1_{Y}\right)\right]=\left[\left(u_{i}⊗v_{j}\right)⊗\left(u_{i^{\prime }}⊗v_{j^{\prime }}\right):i\neq i^{\prime }\;\mathrm{and}\;j=j^{\prime }\right]$;
- $\left[qudit\left(1_{Y}|1_{X}\right)\right]=\left[qudit\left(1_{Y}\right)-qudit\left(1_{X}\right)\right]=\left[\left(u_{i}⊗v_{j}\right)⊗\left(u_{i^{\prime }}⊗v_{j^{\prime }}\right):i=i^{\prime }\;\mathrm{and}\;j\neq j^{\prime }\right]$; and
- $\left[qudit\left(1_{Y}\&1_{X}\right)\right]=\left[qudit\left(1_{Y}\right)\cap qudit\left(1_{X}\right)\right]=\left[\left(u_{i}⊗v_{j}\right)⊗\left(u_{i^{\prime }}⊗v_{j^{\prime }}\right):i\neq i^{\prime }\;\mathrm{and}\;j\neq j^{\prime }\right]$.

Then, as qudit sets: $qudit\left(1_{X},1_{Y}\right)=qudit\left(1_{X}|1_{Y}\right)⊎qudit\left(1_{Y}|1_{X}\right)⊎qudit\left(1_{Y}\&1_{X}\right)$, and the corresponding qudit subspaces stand in the same relation where the disjoint union is replaced by the disjoint sum.

The density operator ρ is represented by the diagonal density matrix $\rho_{X}$ in its own ON basis *X* with ${\left(\rho_{X}\right)}_{ii}=\lambda_{i}$ and similarly for the diagonal density matrix $\tau_{Y}$ with ${\left(\tau_{Y}\right)}_{jj}=\mu_{j}$. The density operators ρ,τ on *V* define a density operator ρ⊗τ on V⊗V with the ON basis of eigenvectors ${\left\{u_{i}⊗v_{j}\right\}}_{i,j}$ and the eigenvalue-probabilities of ${\left\{\lambda_{i}\mu_{j}\right\}}_{i,j}$. The operator ρ⊗τ is represented in its ON basis by the diagonal density matrix $\rho_{X}⊗\tau_{Y}$ with diagonal entries $\lambda_{i}\mu_{j}$ where $1=\left(\lambda_{1}+\cdots +\lambda_{n}\right)\left(\mu_{1}+\cdots +\mu_{n}\right)=\sum_{i,j=1}^{n}\lambda_{i}\mu_{j}$. The probability measure $p\left(u_{i}⊗v_{j}\right)=\lambda_{i}\mu_{j}$ on V⊗V defines the product measure p×p on ${\left(V⊗V\right)}^{2}$ where it can be applied to the qudit subspaces to define the quantum logical entropies as usual.

In the first instance, we have:

$$h\left(1_{X}:\rho ⊗\tau \right)=p\times p\left(\left[qudit\left(1_{X}\right)\right]\right)=\sum \left\{\lambda_{i}\mu_{j}\lambda_{i^{\prime }}\mu_{j^{\prime }}:i\neq i^{\prime }\right\}\;=\sum_{i\neq i^{\prime }}\lambda_{i}\lambda_{i^{\prime }}\sum_{j,j^{\prime }}\mu_{j}\mu_{j^{\prime }}=\sum_{i\neq i^{\prime }}\lambda_{i}\lambda_{i^{\prime }}=1-\sum_{i}\lambda_{i}^{2}=1-\mathrm{tr}\left[\rho^{2}\right]=h\left(\rho \right)$$

and similarly $h\left(1_{Y}:\rho ⊗\tau \right)=h\left(\tau \right)$. Since all the data are supplied by the two density operators, we can use simplified notation to define the corresponding joint, conditional and mutual entropies:

- $h\left(\rho ,\tau \right)=h\left(1_{X},1_{Y}:\rho ⊗\tau \right)=p\times p\left(\left[qudit\left(1_{X}\right)\cup qudit\left(1_{Y}\right)\right]\right)$;
- $h\left(\rho |\tau \right)=h\left(1_{X}|1_{Y}:\rho ⊗\tau \right)=p\times p\left(\left[qudit\left(1_{X}\right)-qudit\left(1_{Y}\right)\right]\right)$;
- $h\left(\tau |\rho \right)=h\left(1_{Y}|1_{X}:\rho ⊗\tau \right)=p\times p\left(\left[qudit\left(1_{Y}\right)-qudit\left(1_{X}\right)\right]\right)$; and
- $m\left(\rho ,\tau \right)=h\left(1_{Y}\&1_{X}:\rho ⊗\tau \right)=p\times p\left(\left[qudit\left(1_{Y}\right)\cap qudit\left(1_{X}\right)\right]\right)$.

Then, the usual Venn diagram relationships hold for the probability measure p×p on ${\left(V⊗V\right)}^{2}$, e.g.,

$$h\left(\rho ,\tau \right)=h\left(\rho |\tau \right)+h\left(\tau |\rho \right)+m\left(\rho ,\tau \right),$$

and probability interpretations are readily available. There are two probability distributions $\lambda ={\left\{\lambda_{i}\right\}}_{i}$ and $\mu ={\left\{\mu_{j}\right\}}_{j}$ on the sample space $\left\{1,\cdots ,n\right\}$. Two pairs $\left(i,j\right)$ and $\left(i^{\prime },j^{\prime }\right)$ are drawn with replacement; the first entry in each pair is drawn according to λ and the second entry according to μ. Then, $h\left(\rho ,\tau \right)$ is the probability that $i\neq i^{\prime }$ or $j\neq j^{\prime }$ (or both); $h\left(\rho |\tau \right)$ is the probability that $i\neq i^{\prime }$ and $j=j^{\prime }$, and so forth. Note that this interpretation assumes no special significance to a $\lambda_{i}$ and $\mu_{i}$ having the same index since we are drawing a pair of pairs.

In the classical case of two probability distributions $p=\left(p_{1},\cdots ,p_{n}\right)$ and $q=\left(q_{1},\cdots ,q_{n}\right)$ on the same index set, the logical cross-entropy is defined as: $h\left(p||q\right)=1-\sum_{i}p_{i}q_{i}$ and interpreted as the probability of getting different indices in drawing a single pair, one from *p* and the other from *q*. However, this cross-entropy assumes some special significance to $p_{i}$ and $q_{i}$ having the same index. However, in our current quantum setting, there is no correlation between the two sets of “index” states ${\left\{u_{i}\right\}}_{i=1,\cdots ,n}$ and ${\left\{v_{j}\right\}}_{j=1,\cdots ,n}$. However, when the two density operators commute, τρ=ρτ, then we can take ${\left\{u_{i}\right\}}_{i=1,\cdots ,n}$ as an ON basis of simultaneous eigenvectors for the two operators with respective eigenvalues $\lambda_{i}$ and $\mu_{i}$ for $u_{i}$ with i=1,⋯,n. In that special case, we can meaningfully define the quantum logical cross-entropy as $h\left(\rho ||\tau \right)=1-\sum_{i=1}^{n}\lambda_{i}\mu_{i}$, but the general case awaits further analysis below.

## 10. The Logical Hamming Distance between Two Partitions

The development of logical quantum information theory in terms of some given commuting or non-commuting observables gives an analysis of the distinguishability of quantum states using those observables. Without any given observables, there is still a natural logical analysis of the distance between quantum states that generalizes the “classical” logical distance $h\left(\pi |\sigma \right)+h\left(\sigma |\pi \right)$ between partitions on a set. In the classical case, we have the logical entropy $h\left(\pi \right)$ of a partition where the partition plays the role of the direct-sum decomposition of eigenspaces of an observable in the quantum case. However, we also have just the logical entropy $h\left(p\right)$ of a probability distribution $p=\left(p_{1},\cdots ,p_{n}\right)$ and the related compound notions of logical entropy given another probability distribution $q=\left(q_{1},\cdots ,q_{n}\right)$ indexed by the same set.

First, we review that classical treatment to motivate the quantum version of the logical Hamming distance between states. A binary relation R⊆U×U on $U=\left\{u_{1},\cdots ,u_{n}\right\}$ can be represented by an n×n *incidence matrix* I(R) where:

$$I{\left(R\right)}_{ij}=\left\{\begin{array}{c} 1\;\mathrm{if}\;\left(u_{i},u_{j}\right)\in R\\ 0\;\mathrm{if}\;\left(u_{i},u_{j}\right)\notin R. \end{array}\right.$$

Taking *R* as the equivalence relation $\mathrm{indit}\left(\pi \right)$ associated with a partition $\pi =\left\{B_{1},\cdots ,B_{I}\right\}$, the *density matrix* $\rho \left(\pi \right)$ *of the partition* π (with equiprobable points) is just the incidence matrix $I\left(\mathrm{indit}\left(\pi \right)\right)$ rescaled to be of trace one (i.e., the sum of diagonal entries is one):

$$\rho \left(\pi \right)=\frac{1}{\left|U\right|}I\left(\mathrm{indit}\left(\pi \right)\right).$$

From coding theory ([31] p. 66), we have the notion of the *Hamming distance between two* 0,1 *vectors or matrices* (of the same dimensions), which is the number of places where they differ. Since logical information theory is about distinctions and differences, it is important to have a classical and quantum logical notion of Hamming distance. The powerset $℘\left(U\times U\right)$ can be viewed as a vector space over $Z_{2}$ where the sum of two binary relations $R,R^{\prime }\subseteq U\times U$ is the symmetric difference, symbolized as $R\Delta R^{\prime }=\left(R-R^{\prime }\right)\cup \left(R^{\prime }-R\right)=R\cup R^{\prime }-R\cap R^{\prime }$, which is the set of elements (i.e., ordered pairs $\left(u_{i},u_{j}\right)\in U\times U$) that are in one set or the other, but not both. Thus, the Hamming distance $D_{H}\left(I\left(R\right),I\left(R^{\prime }\right)\right)$ between the incidence matrices of two binary relations is just the cardinality of their symmetric difference: $D_{H}\left(I\left(R\right),I\left(R^{\prime }\right)\right)=\left|R\Delta R^{\prime }\right|$. Moreover, the size of the symmetric difference does not change if the binary relations are replaced by their complements: $\left|R\Delta R^{\prime }\right|=\left|\left(U^{2}-R\right)\Delta \left(U^{2}-R^{\prime }\right)\right|$.

Hence, given two partitions $\pi =\left\{B_{1},\cdots ,B_{I}\right\}$ and $\sigma =\left\{C_{1},\cdots ,C_{J}\right\}$ on *U*, the unnormalized Hamming distance between the two partitions is naturally defined as (this is investigated in Rossi [32]):

$$D\left(\pi ,\sigma \right)=D_{H}\left(I\left(\mathrm{indit}\left(\pi \right)\right),I\left(\mathrm{indit}\left(\sigma \right)\right)\right)=\left|\mathrm{indit}\left(\pi \right)\Delta \mathrm{indit}\left(\sigma \right)\right|=\left|\mathrm{dit}\left(\pi \right)\Delta \mathrm{dit}\left(\sigma \right)\right|,$$

and the *Hamming distance between* π and σ is defined as the normalized $D\left(\pi ,\sigma \right)$:

$$d\left(\pi ,\sigma \right)=\frac{D\left(\pi ,\sigma \right)}{\left|U\times U\right|}=\frac{\left|\mathrm{dit}\left(\pi \right)\Delta \mathrm{dit}\left(\sigma \right)\right|}{\left|U\times U\right|}=\frac{\left|\mathrm{dit}\left(\pi \right)-\mathrm{dit}\left(\sigma \right)\right|}{\left|U\times U\right|}+\frac{\left|\mathrm{dit}\left(\sigma \right)-\mathrm{dit}\left(\pi \right)\right|}{\left|U\times U\right|}=h\left(\pi |\sigma \right)+h\left(\sigma |\pi \right).$$

This motivates the general case of point probabilities $p=\left(p_{1},\cdots ,p_{n}\right)$ where we define the *Hamming distance* between the two partitions as the sum of the two logical conditional entropies:

$$d\left(\pi ,\sigma \right)=h\left(\pi |\sigma \right)+h\left(\sigma |\pi \right)=2h\left(\pi \vee \sigma \right)-h\left(\pi \right)-h\left(\sigma \right).$$

To motivate the bridge to the quantum version of the Hamming distance, we need to calculate it using the density matrices $\rho \left(\pi \right)$ and $\rho \left(\sigma \right)$ of the two partitions. To compute the trace $\mathrm{tr}\left[\rho \left(\pi \right)\rho \left(\sigma \right)\right]$, we compute the diagonal elements in the product $\rho \left(\pi \right)\rho \left(\sigma \right)$ and add them up: ${\left[\rho \left(\pi \right)\rho \left(\sigma \right)\right]}_{kk}=\sum_{l}\rho {\left(\pi \right)}_{kl}\rho {\left(\sigma \right)}_{lk}=\sum_{l}\sqrt{p_{k}p_{l}}\sqrt{p_{l}p_{k}}$ where the only nonzero terms are where $u_{k},u_{l}\in B\cap C$ for some B∈π and C∈σ. Thus, if $u_{k}\in B\cap C$, then ${\left[\rho \left(\pi \right)\rho \left(\sigma \right)\right]}_{kk}=\sum_{u_{l}\in B\cap C}p_{k}p_{l}$. Therefore, the diagonal element for $u_{k}$ is the sum of the $p_{k}p_{l}$ for $u_{l}$ in the same intersection B∩C as $u_{k}$, so it is $p_{k}\mathrm{Pr}\left(B\cap C\right)$. Then, when we sum over the diagonal elements, then for all the $u_{k}\in B\cap C$ for any given B,C, we just sum $\sum_{u_{k}\in B\cap C}p_{k}\mathrm{Pr}\left(B\cap C\right)=\mathrm{Pr}{\left(B\cap C\right)}^{2}$ so that $\mathrm{tr}\left[\rho \left(\pi \right)\rho \left(\sigma \right)\right]=\sum_{B\in \pi ,C\in \sigma }\mathrm{Pr}{\left(B\cap C\right)}^{2}=1-h\left(\pi \vee \sigma \right)$.

Hence, if we define the *logical cross-entropy* of π and σ as:

$$h\left(\pi ||\sigma \right)=1-\mathrm{tr}\left[\rho \left(\pi \right)\rho \left(\sigma \right)\right],$$

then for partitions on *U* with the point probabilities $p=\left(p_{1},\cdots ,p_{n}\right)$, the logical cross-entropy $h\left(\pi ||\sigma \right)$ of two partitions is the same as the logical joint entropy, which is also the logical entropy of the join:

$$h\left(\pi ||\sigma \right)=h\left(\pi ,\sigma \right)=h\left(\pi \vee \sigma \right).$$

Thus, we can also express the logical Hamming distance between two partitions entirely in terms of density matrices:

$$d\left(\pi ,\sigma \right)=2h\left(\pi ||\sigma \right)-h\left(\pi \right)-h\left(\sigma \right)=\mathrm{tr}\left[\rho {\left(\pi \right)}^{2}\right]+\mathrm{tr}\left[\rho {\left(\sigma \right)}^{2}\right]-2\mathrm{tr}\left[\rho \left(\pi \right)\rho \left(\sigma \right)\right].$$

## 11. The Quantum Logical Hamming Distance

The quantum logical entropy $h\left(\rho \right)=1-\mathrm{tr}\left[\rho^{2}\right]$ of a density matrix ρ generalizes the classical $h\left(p\right)=1-\sum_{i}p_{i}^{2}$ for a probability distribution $p=\left(p_{1},\ldots ,p_{n}\right)$. As a self-adjoint operator, a density matrix has a spectral decomposition $\rho =\sum_{i=1}^{n}\lambda_{i}|u_{i}\rangle \langle u_{i}|$ where ${\left\{|u_{i}\rangle \right\}}_{i=1,\cdots ,n}$ is an orthonormal basis for *V* and where all the eigenvalues $\lambda_{i}$ are real, non-negative and $\sum_{i=1}^{n}\lambda_{i}=1$. Then, $h\left(\rho \right)=1-\sum_{i}\lambda_{i}^{2}$ so $h\left(\rho \right)$ can be interpreted as the probability of getting distinct indices $i\neq i^{\prime }$ in two independent measurements of the state ρ with $\left\{|u_{i}\rangle \right\}$ as the measurement basis. Classically, it is the two-draw probability of getting distinct indices in two independent samples of the probability distribution $\lambda =\left(\lambda_{1},\ldots ,\lambda_{n}\right)$, just as $h\left(p\right)$ is the probability of getting distinct indices in two independent draws on *p*. For a pure state ρ, there is one $\lambda_{i}=1$ with the others zero, and $h\left(\rho \right)=0$ is the probability of getting distinct indices in two independent draws on $\lambda =\left(0,\ldots ,0,1,0,\ldots ,0\right)$.

In the classical case of the logical entropies, we worked with the ditsets or sets of distinctions of partitions. However, everything could also be expressed in terms of the complementary sets of indits or indistinctions of partitions (ordered pairs of elements in the same block of the partition) since: $\mathrm{dit}\left(\pi \right)⊎\mathrm{indit}\left(\pi \right)=U\times U$. When we switch to the density matrix treatment of “classical” partitions, then the focus shifts to the indistinctions. For a partition $\pi =\left\{B_{1},\ldots ,B_{I}\right\}$, the logical entropy is the sum of the distinction probabilities: $h\left(\pi \right)=\sum_{\left(u_{k},u_{l}\right)\in \mathrm{dit}\left(\pi \right)}p_{k}p_{l}$, which in terms of indistinctions is:

$$h\left(\pi \right)=1-\sum_{\left(u_{k},u_{l}\right)\in \mathrm{indit}\left(\pi \right)}p_{k}p_{l}=1-\sum_{i=1}^{I}\mathrm{Pr}{\left(B_{i}\right)}^{2}.$$

When expressed in the density matrix formulation, then $\mathrm{tr}\left[\rho {\left(\pi \right)}^{2}\right]$ is the sum of the indistinction probabilities:

$$\mathrm{tr}\left[\rho {\left(\pi \right)}^{2}\right]=\sum_{\left(u_{k},u_{l}\right)\in \mathrm{indit}\left(\pi \right)}p_{k}p_{l}=\sum_{i=1}^{I}\mathrm{Pr}{\left(B_{i}\right)}^{2}.$$

The nonzero entries in $\rho \left(\pi \right)$ have the form $\sqrt{p_{k}p_{l}}$ for $\left(u_{k},u_{l}\right)\in \mathrm{indit}\left(\pi \right)$; their squares are the indistinction probabilities. That provides the interpretive bridge to the quantum case.

The quantum analogue of an indistinction probability is the absolute square ${\left|\rho_{kl}\right|}^{2}$ of a nonzero entry $\rho_{kl}$ in a density matrix ρ, and $\mathrm{tr}\left[\rho^{2}\right]=\sum_{k,l}{\left|\rho_{kl}\right|}^{2}$ is the sum of those “indistinction” probabilities. The nonzero entries in the density matrix ρ might be called “*coherences*” so that $\rho_{kl}$ may be interpreted as the amplitudes for the states $u_{k}$ and $u_{l}$ to cohere together in the state ρ, so $\mathrm{tr}\left[\rho^{2}\right]$ is the sum of the *coherence probabilities*, just as $\mathrm{tr}\left[\rho {\left(\pi \right)}^{2}\right]=\sum_{\left(u_{k},u_{l}\right)\in \mathrm{indit}\left(\pi \right)}p_{k}p_{l}$ is the sum of the indistinction probabilities. The quantum logical entropy $h\left(\rho \right)=1-\mathrm{tr}\left[\rho^{2}\right]$ may then be interpreted as the sum of the decoherence probabilities, just as $h\left(\rho \left(\pi \right)\right)=h\left(\pi \right)=1-\sum_{\left(u_{k},u_{l}\right)\in \mathrm{indit}\left(\pi \right)}p_{k}p_{l}$ is the sum of the distinction probabilities.

This motivates the general quantum definition of the joint entropy $h\left(\pi ,\sigma \right)=h\left(\pi \vee \sigma \right)=h\left(\pi ||\sigma \right)$, which is the:

$$h\left(\rho ||\tau \right)=1-\mathrm{tr}\left[\rho^{†}\tau \right]\;\;\mathrm{quantum}\;\mathrm{logical}\;\mathrm{cross-entropy}.$$

To work out its interpretation, we again take ON eigenvector bases ${\left\{|u_{i}\rangle \right\}}_{i=1}^{n}$ for ρ and ${\left\{|v_{j}\rangle \right\}}_{j=1}^{n}$ for τ with $\lambda_{i}$ and $\mu_{j}$ as the respective eigenvalues and compute the operation of $\tau^{†}\rho :V\to V$. Now, $|u_{i}\rangle =\sum_{j}\langle v_{j}|u_{i}\rangle |v_{j}\rangle$ so $\rho |u_{i}\rangle =\lambda_{i}|u_{i}\rangle =\sum_{j}\lambda_{i}\langle v_{j}|u_{i}\rangle |v_{j}\rangle$, and then, for $\tau^{†}=\sum_{j}|v_{j}\rangle \langle v_{j}|\mu_{j}$, so $\tau^{†}\rho |u_{i}\rangle =\sum_{j}\lambda_{i}\mu_{j}\langle v_{j}|u_{i}\rangle |v_{j}\rangle$. Thus, $\tau^{†}\rho$ in the ${\left\{u_{i}\right\}}_{i}$ basis would have the diagonal entries $\langle u_{i}\left|\tau^{†}\rho \right|u_{i}\rangle =\sum_{j}\lambda_{i}\mu_{j}\langle v_{j}|u_{i}\rangle \langle u_{i}|v_{j}\rangle$, so the trace is:

$$\mathrm{tr}\left[\tau^{†}\rho \right]=\sum_{i}\langle u_{i}\left|\tau^{†}\rho \right|u_{i}\rangle =\sum_{i,j}\lambda_{i}\mu_{j}\langle v_{j}|u_{i}\rangle \langle u_{i}|v_{j}\rangle =\mathrm{tr}\left[\rho^{†}\tau \right]$$

which is symmetrical. The other information we have is the $\sum_{i}\lambda_{i}=1=\sum_{j}\mu_{j}$, and they are non-negative. The classical logical cross-entropy of two probability distributions is $h\left(p||q\right)=1-\sum_{i,j}p_{i}q_{j}\delta_{ij}$, where two indices *i* and *j* are either identical or totally distinct. However, in the quantum case, the “index” states $|u_{i}\rangle$ and $|v_{j}\rangle$ have an “overlap” measured by the inner product $\langle u_{i}|v_{j}\rangle$. The trace $\mathrm{tr}\left[\rho^{†}\tau \right]$ is real since $\langle v_{j}|u_{i}\rangle ={\langle u_{i}|v_{j}\rangle }^{*}$ and $\langle v_{j}|u_{i}\rangle \langle u_{i}|v_{j}\rangle ={\left|\langle u_{i}|v_{j}\rangle \right|}^{2}={\left|\langle v_{j}|u_{i}\rangle \right|}^{2}$ is the probability of getting $\lambda_{i}$ when measuring $v_{j}$ in the $u_{i}$ basis and the probability of getting $\mu_{j}$ when measuring $u_{i}$ in the $v_{j}$ basis. The twofold nature of density matrices as states and as observables then allows $\mathrm{tr}\left[\rho^{†}\tau \right]$ to be interpreted as the average value of the observable ρ when measuring the state τ or vice versa.

We may call $\langle v_{j}|u_{i}\rangle \langle u_{i}|v_{j}\rangle$ the *proportion* or *extent of overlap* for those two index states. Thus, $\mathrm{tr}\left[\rho^{†}\tau \right]$ is the sum of all the probability combinations $\lambda_{i}\mu_{j}$ weighted by the overlaps $\langle v_{j}|u_{i}\rangle \langle u_{i}|v_{j}\rangle$ for the index states $|u_{i}\rangle$ and $|v_{j}\rangle$. The quantum logical cross-entropy can be written in a number of ways:

$$h\left(\rho ||\tau \right)=1-\mathrm{tr}\left[\rho^{†}\tau \right]=1-\sum_{i,j}\lambda_{i}\mu_{j}\langle v_{j}|u_{i}\rangle \langle u_{i}|v_{j}\rangle \;=\mathrm{tr}\left[\tau^{†}\left(I-\rho \right)\right]=\sum_{i,j}\left(1-\lambda_{i}\right)\mu_{j}\langle v_{j}|u_{i}\rangle \langle u_{i}|v_{j}\rangle \;=\mathrm{tr}\left[\rho^{†}\left(I-\tau \right)\right]=\sum_{i,j}\lambda_{i}\left(1-\mu_{j}\right)\langle v_{j}|u_{i}\rangle \langle u_{i}|v_{j}\rangle$$.

Classically, the “index state” $\left\{i\right\}$ completely overlaps with $\left\{j\right\}$ when i=j and has no overlap with any other $\left\{i^{\prime }\right\}$ from the indices $\left\{1,\ldots ,n\right\}$, so the “overlaps” are, as it were, $\langle j|i\rangle \langle i|j\rangle =\delta_{ij}$, the Kronecker delta. Hence, the classical analogue formulas are:

$$h\left(p||q\right)=1-\sum_{i,j}p_{i}q_{j}\delta_{ij}=\sum_{i,j}\left(1-p_{i}\right)q_{j}\delta_{ij}=\sum_{i,j}p_{i}\left(1-q_{j}\right)\delta_{ij}.$$

The quantum logical cross-entropy $h\left(\rho ||\tau \right)$ can be interpreted by considering two measurements, one of ρ with the ${\left\{|u_{i}\rangle \right\}}_{i}$ measurement basis and the other of τ with the ${\left\{|v_{j}\rangle \right\}}_{j}$ measurement basis. If the outcome of the ρ measurement was $u_{i}$ with probability $\lambda_{i}$, then the outcome of the τ measurement is different than $v_{j}$ with probability $1-\mu_{j}$; however, that distinction probability $\lambda_{i}\left(1-\mu_{j}\right)$ is only relevant to the extent that $u_{i}$ and $v_{j}$ are the “same state” or overlap, and that extent is $\langle v_{j}|u_{i}\rangle \langle u_{i}|v_{j}\rangle$. Hence, the quantum logical cross-entropy is the sum of those two-measurement distinction probabilities weighted by the extent that the states overlap. The interpretation of $h\left(\rho \right)$ and $h\left(\tau ||\rho \right)$, as well as the later development of the quantum logical conditional entropy $h\left(\rho |\tau \right)$ and the quantum Hamming distance $d\left(\rho ,\tau \right)$ are all based on using the eigenvectors and eigenvalues of density matrices, which Michael Nielsen and Issac Chuang seem to prematurely dismiss as having little or no “special significance” ([30] p. 103).

When the two density matrices commute, ρτ=τρ, then (as noted above) we have the essentially classical situation of one set of index states ${\left\{|u_{i}\rangle \right\}}_{i}$ which is an orthonormal basis set of simultaneous eigenvectors for both ρ and τ with the respective eigenvalues ${\left\{\lambda_{i}\right\}}_{i}$ and ${\left\{\mu_{j}\right\}}_{j}$. Then, $\langle u_{j}|u_{i}\rangle \langle u_{i}|u_{j}\rangle =\delta_{ij}$, so $h\left(\rho ||\tau \right)=\sum_{i,j}\lambda_{i}\left(1-\mu_{j}\right)\delta_{ij}$ is the probability of getting two distinct index states $u_{i}$ and $u_{j}$ for i≠j in two independent measurements, one of ρ and one of τ in the same measurement basis of ${\left\{|u_{i}\rangle \right\}}_{i}$. This interpretation includes the special case when τ=ρ and $h\left(\rho ||\rho \right)=h\left(\rho \right)$.

We saw that classically, the logical Hamming distance between two partitions could be defined as:

$$d\left(\pi ,\sigma \right)=2h\left(\pi ||\sigma \right)-h\left(\pi \right)-h\left(\sigma \right)=\mathrm{tr}\left[\rho {\left(\pi \right)}^{2}\right]+\mathrm{tr}\left[\rho {\left(\sigma \right)}^{2}\right]-2\mathrm{tr}\left[\rho \left(\pi \right)\rho \left(\sigma \right)\right]$$

so this motivates the quantum definition. Nielsen and Chuang suggest the idea of a Hamming distance between quantum states, only to then dismiss it. “Unfortunately, the Hamming distance between two objects is simply a matter of labeling, and a priori there are not any labels in the Hilbert space arena of quantum mechanics!” ([30] p. 399). They are right that there is no correlation, say, between the vectors in the two ON bases ${\left\{u_{i}\right\}}_{i}$ and ${\left\{v_{j}\right\}}_{j}$ for *V*, but the cross-entropy $h\left(\rho ||\tau \right)$ uses all possible combinations in the terms $\lambda_{i}\left(1-\mu_{j}\right)\langle v_{j}|u_{i}\rangle \langle u_{i}|v_{j}\rangle$; thus, the definition of the Hamming distance given below does not use any arbitrary labeling or correlations.

$$d\left(\rho ,\tau \right)=2h\left(\rho ||\tau \right)-h\left(\rho \right)-h\left(\tau \right)=\mathrm{tr}\left[\rho^{2}\right]+\mathrm{tr}\left[\tau^{2}\right]-2\mathrm{tr}\left[\rho^{†}\tau \right]$$

This is the definition of the *quantum logical Hamming distance* between two quantum states.

There is another distance measure between quantum states, namely the Hilbert–Schmidt norm, which has been recently investigated in [29] (with an added $\frac{1}{2}$ factor). It is the square of the Euclidean distance between the quantum states, and ignoring the $\frac{1}{2}$ factor, it is the square of the “trace distance” ([30] Chapter 9) between the states.

$$\mathrm{tr}\left[{\left(\rho -\tau \right)}^{2}\right]\;\;\mathrm{Hilbert-chmidt}\;\mathrm{norm},$$

where we write $A^{2}$ for $A^{†}A$. Then, the naturalness of this norm as a “distance” is enhanced by the fact that it is the same as the quantum Hamming distance:

**Theorem** **4.** *Hilbert–Schmidt norm = quantum logical Hamming distance.*

**Proof.**  $\mathrm{tr}\left[{\left(\rho -\tau \right)}^{2}\right]=\mathrm{tr}\left[\rho^{2}\right]+\mathrm{tr}\left[\tau^{2}\right]-2\mathrm{tr}\left[\rho^{†}\tau \right]=2h\left(\rho ||\tau \right)-h\left(\rho \right)-h\left(\tau \right)=d\left(\rho ,\tau \right).$ ☐

Hence, the *information inequality* holds trivially for the quantum logical Hamming distance:

$$d\left(\rho ,\tau \right)\geq 0\;\mathrm{with}\;\mathrm{equality}\;\mathrm{iff}\;\rho =\tau .$$

The fundamental theorem can be usefully restated in this broader context of density operators instead of in terms of the density matrix represented in the ON basis for *F*-eigenvectors. Let ρ be any state to be measured by an observable *F*, and let $\hat{\rho }=\sum_{i=1}^{I}P_{\phi_{i}}\rho P_{\phi_{i}}$ be the result of applying the Lüders mixture operation (where the $P_{\phi_{i}}$ are the projection operators to the eigenspaces of *F*). Then, a natural question to ask is the Hilbert–Schmidt norm or quantum logical Hamming distance between the pre- and post-measurement states. It might be noted that the Hilbert–Schmidt norm and the Lüders mixture operation are defined independently of any considerations of logical entropy.

**Theorem** **5** (**Fundamental** **(quantum)**)**.** *$\mathrm{tr}\left[{\left(\rho -\hat{\rho }\right)}^{2}\right]=h\left(\hat{\rho }\right)-h\left(\rho \right)$.*

**Proof.**  $\mathrm{tr}\left[{\left(\rho -\hat{\rho }\right)}^{2}\right]=\mathrm{tr}\left[\left(\rho^{†}-{\hat{\rho }}^{†}\right)\left(\rho -\hat{\rho }\right)\right]=\mathrm{tr}\left[\rho^{2}\right]+\mathrm{tr}\left[{\hat{\rho }}^{2}\right]-\mathrm{tr}\left[\rho \hat{\rho }\right]-\mathrm{tr}\left[\hat{\rho }\rho \right]$ where $\rho^{†}=\rho$, ${\hat{\rho }}^{†}=\hat{\rho }$, $\mathrm{tr}\left[\rho \hat{\rho }\right]=\mathrm{tr}\left[\hat{\rho }\rho \right]$ and $\mathrm{tr}\left[\rho \hat{\rho }\right]=\mathrm{tr}\left[\rho \sum_{i=1}^{I}P_{\phi_{i}}\rho P_{\phi_{i}}\right]=\mathrm{tr}\left[\sum \rho P_{\phi_{i}}\rho P_{\phi_{i}}\right]$. Also $\mathrm{tr}\left[{\hat{\rho }}^{2}\right]=\mathrm{tr}\left[\left(\sum_{i}P_{\phi_{i}}\rho P_{\phi_{i}}\right)\left(\sum_{j}P_{\phi_{j}}\rho P_{\phi_{j}}\right)\right]=\mathrm{tr}\left[\sum_{i}P_{\phi_{i}}\rho P_{\phi_{i}}^{2}\rho P_{\phi_{i}}\right]$ using the orthogonality of the distinct projection operators. Then, using the idempotency of the projections and the cyclicity of the trace, $\mathrm{tr}\left[{\hat{\rho }}^{2}\right]=\mathrm{tr}\left[\sum_{i}\rho P_{\phi_{i}}\rho P_{\phi_{i}}\right]$ so $\mathrm{tr}\left[\rho \hat{\rho }\right]=\mathrm{tr}\left[\hat{\rho }\rho \right]=\mathrm{tr}\left[{\hat{\rho }}^{2}\right]$, and hence, $\mathrm{tr}\left[{\left(\rho -\hat{\rho }\right)}^{2}\right]=\mathrm{tr}\left[\rho^{2}\right]-\mathrm{tr}\left[{\hat{\rho }}^{2}\right]=h\left(\hat{\rho }\right)-h\left(\rho \right)$. ☐

## 12. Results

Logical information theory arises as the quantitative version of the logic of partitions just as logical probability theory arises as the quantitative version of the dual Boolean logic of subsets. Philosophically, logical information is based on the idea of information-as-distinctions. The Shannon definitions of entropy arise naturally out of the logical definitions by replacing the counting of distinctions by the counting of the minimum number of binary partitions (bits) that are required, on average, to make all the same distinctions, i.e., to encode the distinguished elements uniquely, which is why the Shannon theory is so well adapted for the theory of coding and communication.

This “classical” logical information theory may be developed with the data of two partitions on a set with point probabilities. Section 7 gives the generalization to the quantum case where the partitions are provided by two commuting observables (the point set is an ON basis of simultaneous eigenvectors), and the point probabilities are provided by the state to be measured. In Section 8, the fundamental theorem for quantum logical entropy and measurement established a direct quantitative connection between the increase in quantum logical entropy due to a projective measurement and the eigenstates (cohered together in the pure superposition state being measured) that are distinguished by the measurement (decohered in the post-measurement mixed state). This theorem establishes quantum logical entropy as a natural notion for a quantum information theory focusing on distinguishing states.

The classical theory might also start with partitions on two different sets and a probability distribution on the product of the sets (see Appendix A). Appendix B gives the quantum generalization of that case with the two sets being two ON bases for two non-commuting observables, and the probabilities are provided by a state to be measured. The classical theory may also be developed just using two probability distributions indexed by the same set, and this is generalized to the quantum case where we are just given two density matrices representing two states in a Hilbert space. Section 10 and Section 11 carry over the Hamming distance measure from the classical to the quantum case where it is equal to the Hilbert–Schmidt distance (square of the trace distance). The general fundamental theorem relating measurement and logical entropy is that the Hilbert–Schmidt distance (=quantum logical Hamming distance) between any pre-measurement state ρ and the state $\hat{\rho }$ resulting from a projective measurement of the state is the difference in their logical entropies, $h\left(\hat{\rho }\right)-h\left(\rho \right)$.

## 13. Discussion

The overall argument is that quantum logical entropy is the simple and natural notion of information-as-distinctions for quantum information theory focusing on the distinguishing of quantum states. These results add to the arguments already presented by Manfredi and Feix [21] and many others (see [23]) for this notion of quantum entropy.

There are two related classical theories of information, classical logical information theory (focusing on information-as-distinctions and analyzing classification) and the Shannon theory (focusing on coding and communications theory). Generalizing to the quantum case, there are also two related quantum theories of information, the logical theory (using quantum logical entropy to focus on distinguishing quantum states and analyzing measurement as the quantum version of classification) and the conventional quantum information theory (using von Neumann entropy to develop a quantum version of the Shannon treatment of coding and communications).

## Figures and Tables

**Figure A1:**
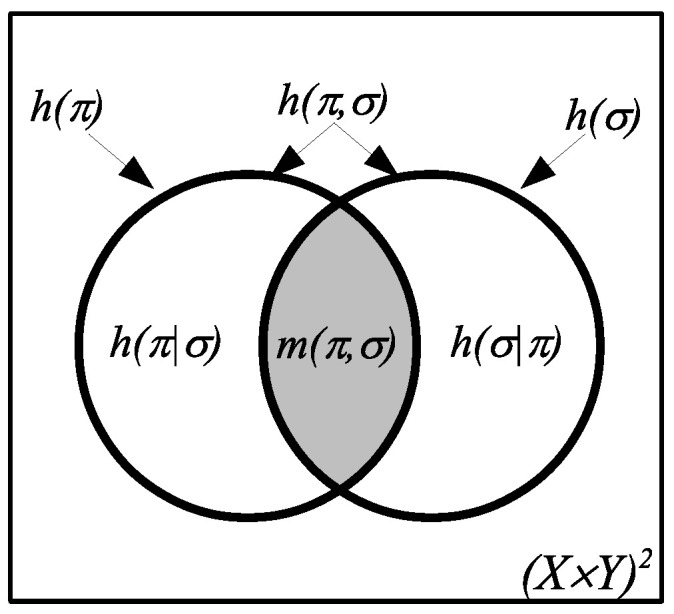
Venn diagram for logical entropies as values of a probability measure p×p on ${\left(X\times Y\right)}^{2}$.

**Figure A2:**
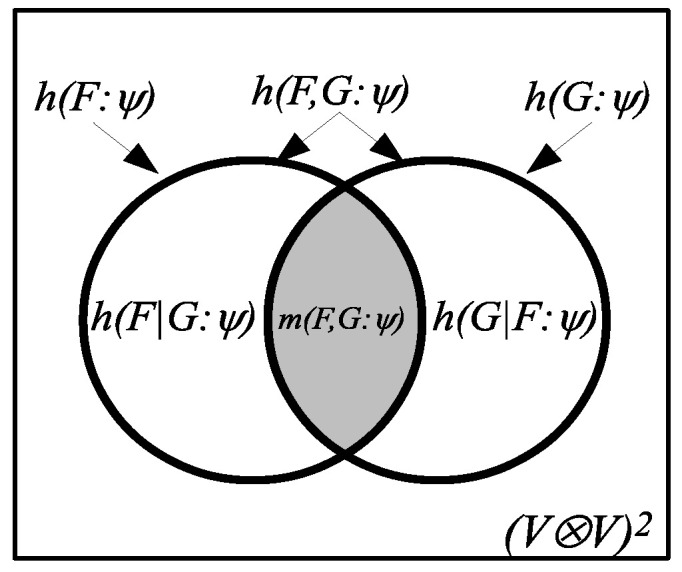
Venn diagram for quantum logical entropies as probabilities on ${\left(V⊗V\right)}^{2}$.

## Appendix A. Classical Logical Information Theory with Two Sets *X* and *Y*

The usual (“classical”) logical information theory for a probability distribution $\left\{p\left(x,y\right)\right\}$ on X×Y (finite) in effect uses the discrete partition on *X* and *Y* [3]. For the general case of quantum logical entropy for not-necessarily commuting observables, we need to first briefly develop the classical case with general partitions on *X* and *Y*.

Given two finite sets *X* and *Y* and real-valued functions f:X→R with values ${\left\{\phi_{i}\right\}}_{i=1}^{I}$ and g:Y→R with values ${\left\{\gamma_{j}\right\}}_{j=1}^{J}$, each function induces a partition on its domain:

$$\pi ={\left\{f^{-1}\left(\phi_{i}\right)\right\}}_{i\in I}=\left\{B_{1},\cdots ,B_{I}\right\}\;\mathrm{on}\;X,\;\mathrm{and}\;\sigma ={\left\{g^{-1}\left(\gamma_{j}\right)\right\}}_{j\in J}=\left\{C_{1},\cdots ,C_{J}\right\}\;\mathrm{on}\;Y.$$

We need to define logical entropies on X×Y, but first, we need to define the ditsets or information sets.

A partition $\pi =\left\{B_{1},\cdots ,B_{I}\right\}$ on *X* and a partition $\sigma =\left\{C_{1},\cdots ,C_{J}\right\}$ on *Y* define a *product partition* π×σ on X×Y whose blocks are ${\left\{B_{i}\times C_{j}\right\}}_{i,j}$. Then, π induces $\pi \times 0_{Y}$ on X×Y (where $0_{Y}$ is the indiscrete partition on *Y*), and σ induces $0_{X}\times \sigma$ on X×Y. The corresponding ditsets or information sets are:

- $\mathrm{dit}\left(\pi \times 0_{Y}\right)=\left\{\left(\left(x,y\right),\left(x^{\prime },y^{\prime }\right)\right):f\left(x\right)\neq f\left(x^{\prime }\right)\right\}\subseteq {\left(X\times Y\right)}^{2}$;
- $\mathrm{dit}\left(0_{X}\times \sigma \right)=\left\{\left(\left(x,y\right),\left(x^{\prime },y^{\prime }\right)\right):g\left(y\right)\neq g\left(y^{\prime }\right)\right\}\subseteq {\left(X\times Y\right)}^{2}$;
- $\mathrm{dit}\left(\pi \times \sigma \right)=\mathrm{dit}\left(\pi \times 0_{Y}\right)\cup \mathrm{dit}\left(0_{X}\times \sigma \right)$; and so forth.

Given a joint probability distribution $p:X\times Y\to \left[0,1\right]$, the product probability distribution is $p\times p:{\left(X\times Y\right)}^{2}\to \left[0,1\right]$.

All the logical entropies are just the product probabilities of the ditsets and their union, differences and intersection:

- $h\left(\pi \times 0_{Y}\right)=p\times p\left(\mathrm{dit}\left(\pi \times 0_{Y}\right)\right)$;
- $h\left(0_{X}\times \sigma \right)=p\times p\left(\mathrm{dit}\left(0_{X}\times \sigma \right)\right)$;
- $h\left(\pi \times \sigma \right)=p\times p\left(\mathrm{dit}\left(\pi \times \sigma \right)\right)=p\times p\left(\mathrm{dit}\left(\pi \times 0_{Y}\right)\cup \mathrm{dit}\left(0_{X}\times \sigma \right)\right)$;
- $h\left(\pi \times 0_{Y}|0_{X}\times \sigma \right)=p\times p\left(\mathrm{dit}\left(\pi \times 0_{Y}\right)-\mathrm{dit}\left(0_{X}\times \sigma \right)\right)$;
- $h\left(0_{X}\times \sigma |\pi \times 0_{Y}\right)=p\times p\left(\mathrm{dit}\left(0_{X}\times \sigma \right)-\mathrm{dit}\left(\pi \times 0_{Y}\right)\right)$;
- $m\left(\pi \times 0_{Y},0_{X}\times \sigma \right)=p\times p\left(\mathrm{dit}\left(\pi \times 0_{Y}\right)\cap \mathrm{dit}\left(0_{X}\times \sigma \right)\right)$.

All the logical entropies have the usual two-draw probability interpretation where the two independent draws from X×Y are $\left(x,y\right)$ and $\left(x^{\prime },y^{\prime }\right)$ and can be interpreted in terms of the *f*-values and *g*-values:

- $h\left(\pi \times 0_{Y}\right)$ = probability of getting distinct *f*-values;
- $h\left(0_{X}\times \sigma \right)$ = probability of getting distinct *g*-values;
- $h\left(\pi \times \sigma \right)$ = probability of getting distinct *f*- or *g*-values;
- $h\left(\pi \times 0_{Y}|0_{X}\times \sigma \right)$ = probability of getting distinct *f*-values, but the same *g*-values;
- $h\left(0_{X}\times \sigma |\pi \times 0_{Y}\right)$ = probability of getting distinct *g*-values, but the same *f*-values;
- $m\left(\pi \times 0_{Y},0_{X}\times \sigma \right)$ = probability of getting distinct *f*- and distinct *g*-values.

We have defined all the logical entropies by the general method of the product probabilities on the ditsets. In the first three cases, $h\left(\pi \times 0_{Y}\right)$, $h\left(0_{X}\times \sigma \right)$ and $h\left(\pi \times \sigma \right)$, they were the logical entropies of partitions on X×Y, so they could equivalently be defined using density matrices. The case of $h\left(\pi \times \sigma \right)$ illustrates the general case. If $\rho \left(\pi \right)$ is the density matrix defined for π on *X* and $\rho \left(\sigma \right)$ the density matrix for σ on *Y*, then $\rho \left(\pi \times \sigma \right)=\rho \left(\pi \right)⊗\rho \left(\sigma \right)$ is the density matrix for π×σ defined on X×Y, and:

$$h\left(\pi \times \sigma \right)=1-\mathrm{tr}\left[\rho {\left(\pi \times \sigma \right)}^{2}\right].$$

The marginal distributions are: $p_{X}\left(x\right)=\sum_{y}p\left(x,y\right)$ and $p_{Y}\left(y\right)=\sum_{x}p\left(x,y\right)$. Since π is a partition on *X*, there is also the usual logical entropy $h\left(\pi \right)=p_{X}\times p_{X}\left(\mathrm{dit}\left(\pi \right)\right)=1-\mathrm{tr}\left[\rho {\left(\pi \right)}^{2}\right]=h\left(\pi \times 0_{Y}\right)$ where $\mathrm{dit}\left(\pi \right)\subseteq X\times X$ and similarly for $p_{Y}$.

Since the context should be clear, we may henceforth adopt the old notation from the case where π and σ were partitions on the same set *U*, i.e., $h\left(\pi \right)=h\left(\pi \times 0_{Y}\right)$, $h\left(\sigma \right)=h\left(0_{X}\times \sigma \right)$, $h\left(\pi ,\sigma \right)=h\left(\pi \times \sigma \right)$, etc.

Since the logical entropies are the values of a probability measure, all the usual identities hold where the underlying set is now ${\left(X\times Y\right)}^{2}$ instead of $U^{2}$, as illustrated in Figure A1.

The previous treatment of $h\left(X\right)$, $h\left(Y\right)$, $h\left(X,Y\right)$, $h\left(X|Y\right)$, $h\left(Y|X\right)$ and $m\left(X,Y\right)$ in [3] was just the special cases where $\pi =1_{X}$ and $\sigma =1_{Y}$.

## Appendix B. Quantum Logical Entropies with Non-Commuting Observables

As before in the case of commuting observables, the quantum case can be developed in close analogy with the previous classical case. Given a finite-dimensional Hilbert space *V* and not necessarily commuting observables F,G:V→V, let *X* be an orthonormal basis of *V* of *F*-eigenvectors, and let *Y* be an orthonormal basis for *V* of *G*-eigenvectors (so $\left|X\right|=\left|Y\right|$).

Let f:X→R be the eigenvalue function for *F* with values ${\left\{\phi_{i}\right\}}_{i=1}^{I}$, and let g:Y→R be the eigenvalue function for *G* with values ${\left\{\gamma_{j}\right\}}_{j=1}^{J}$.

Each eigenvalue function induces a partition on its domain:

$$\pi =\left\{f^{-1}\left(\phi_{i}\right)\right\}=\left\{B_{1},\cdots ,B_{I}\right\}\;\mathrm{on}\;X,\;\mathrm{and}\;\sigma =\left\{g^{-1}\left(\gamma_{j}\right)\right\}=\left\{C_{1},\cdots ,C_{J}\right\}\;\mathrm{on}\;Y.$$

We associated with the ordered pair $\left(x,y\right)$, the basis element x⊗y in the basis ${\left\{x⊗y\right\}}_{x\in X,y\in Y}$ for V⊗V. Then, each pair of pairs $\left(\left(x,y\right),\left(x^{\prime },y^{\prime }\right)\right)$ is associated with the basis element $\left(x⊗y\right)⊗\left(x^{\prime }⊗y^{\prime }\right)$ in $\left(V⊗V\right)⊗\left(V⊗V\right)={\left(V⊗V\right)}^{2}$.

Instead of ditsets or information sets, we now have qudit subspaces or information subspaces. For $R\subseteq {\left(V⊗V\right)}^{2}$, let $\left[R\right]$ be the subspace generated by *R*. We simplify the notation of $qudit\left(\pi \times 0_{Y}\right)=qudit\left(\pi \right)=\left\{\left(x⊗y\right)⊗\left(x^{\prime }⊗y^{\prime }\right):f\left(x\right)\neq f\left(x^{\prime }\right)\right\}$, etc.

- $\left[qudit\left(\pi \right)\right]=\left[\left\{\left(x⊗y\right)⊗\left(x^{\prime }⊗y^{\prime }\right):f\left(x\right)\neq f\left(x^{\prime }\right)\right\}\right]$;
- $\left[qudit\left(\sigma \right)\right]=\left[\left\{\left(x⊗y\right)⊗\left(x^{\prime }⊗y^{\prime }\right):g\left(y\right)\neq g\left(y^{\prime }\right)\right\}\right]$;
- $\left[qudit\left(\pi ,\sigma \right)\right]=\left[qudit\left(\pi \right)\cup qudit\left(\sigma \right)\right]$; and so forth. Tt is again an easy implication of the aforementioned common dits theorem that any two nonzero information spaces $\left[qudit\left(\pi \right)\right]$ and $\left[qudit\left(\sigma \right)\right]$ have a nonzero intersection, so the mutual information space $\left[qudit\left(\pi \right)\cap qudit\left(\sigma \right)\right]$ is not the zero space.

A normalized state $|\psi \rangle$ on V⊗V defines a pure state density matrix $\rho \left(\psi \right)=|\psi \rangle \langle \psi |$. Let $\alpha_{x,y}=\langle x⊗y|\psi \rangle$, so if $P_{\left[x⊗y\right]}$ is the projection to the subspace (ray) generated by x⊗y in V⊗V, then a probability distribution on X×Y is defined by:

$$p\left(x,y\right)=\alpha_{x,y}\alpha_{x,y}^{*}=\mathrm{tr}\left[P_{\left[x⊗y\right]}\rho \left(\psi \right)\right],$$

or more generally, for a subspace T⊆V⊗V, a probability distribution is defined on the subspaces by:

$$\mathrm{Pr}\left(T\right)=\mathrm{tr}\left[P_{T}\rho \left(\psi \right)\right].$$

Then, the product probability distribution p×p on the subspaces of ${\left(V⊗V\right)}^{2}$ defines the quantum logical entropies when applied to the information subspaces:

- $h\left(F:\psi \right)=p\times p\left(\left[qudit\left(\pi \right)\right]\right)=\mathrm{tr}\left[P_{\left[qudit\left(\pi \right)\right]}\left(\rho \left(\psi \right)⊗\rho \left(\psi \right)\right)\right]$;
- $h\left(G:\psi \right)=p\times p\left(\left[qudit\left(\sigma \right)\right]\right)=\mathrm{tr}\left[P_{\left[qudit\left(\sigma \right)\right]}\left(\rho \left(\psi \right)⊗\rho \left(\psi \right)\right)\right]$;
- $h\left(F,G:\psi \right)=p\times p\left(\left[qudit\left(\pi \right)\cup qudit\left(\sigma \right)\right]\right)=\mathrm{tr}\left[P_{\left[qudit\left(\pi \right)\cup qudit\left(\sigma \right)\right]}\left(\rho \left(\psi \right)⊗\rho \left(\psi \right)\right)\right]$;
- $h\left(F|G:\psi \right)=p\times p\left(\left[qudit\left(\pi \right)-qudit\left(\sigma \right)\right]\right)=\mathrm{tr}\left[P_{\left[qudit\left(\pi \right)-qudit\left(\sigma \right)\right]}\left(\rho \left(\psi \right)⊗\rho \left(\psi \right)\right)\right]$;
- $h\left(G|F:\psi \right)=p\times p\left(\left[qudit\left(\sigma \right)-qudit\left(\pi \right)\right]\right)=\mathrm{tr}\left[P_{\left[qudit\left(\sigma \right)-qudit\left(\pi \right)\right]}\left(\rho \left(\psi \right)⊗\rho \left(\psi \right)\right)\right]$;
- $m\left(F,G:\psi \right)=p\times p\left(\left[qudit\left(\pi \right)\cap qudit\left(\sigma \right)\right]\right)=\mathrm{tr}\left[P_{\left[qudit\left(\pi \right)\cap qudit\left(\sigma \right)\right]}\left(\rho \left(\psi \right)⊗\rho \left(\psi \right)\right)\right]$.

The observable F:V→V defines an observable F⊗I:V⊗V→V⊗V with the eigenvectors x⊗v for any nonzero v∈V and with the same eigenvalues $\phi_{1},\cdots ,\phi_{I}$ (the context should suffice to distinguish the identity operator I:V→V from the index set *I* for the *F*-eigenvalues). Then, in two independent measurements of ψ by the observable F⊗I, we have:

$$h\left(F:\psi \right)=\;\mathrm{probability}\;\mathrm{of}\;\mathrm{getting}\;\mathrm{distinct}\;\mathrm{eigenvalues}\phi_{i}\;\mathrm{and}\;\phi_{i^{\prime }},\mathrm{i.e.},\mathrm{of}\;\mathrm{getting}\;a\;\mathrm{qudit}\;\mathrm{of}\;F.$$

In a similar manner, G:V→V defines the observable I⊗G:V⊗V→V⊗V with the eigenvectors v⊗y and with the same eigenvalues $\gamma_{1},\cdots ,\gamma_{J}$. Then, in two independent measurements of ψ by the observable I⊗G, we have:

$$h\left(G:\psi \right)=\;\mathrm{probability}\;\mathrm{of}\;\mathrm{getting}\;\mathrm{distinct}\;\mathrm{eigenvalues}\;\gamma_{j}\;\mathrm{and}\;\gamma_{j^{\prime }}.$$

The two observables F,G:V→V define an observable F⊗G:V⊗V→V⊗V with the eigenvectors x⊗y for $\left(x,y\right)\in X\times Y$ and eigenvalues $f\left(x\right)g\left(y\right)=\phi_{i}\gamma_{j}$. To interpret the compound logical entropies cleanly, we assume there is no accidental degeneracy, so there are no $\phi_{i}\gamma_{j}=\phi_{i^{\prime }}\gamma_{j^{\prime }}$ for $i\neq i^{\prime }$ and $j\neq j^{\prime }$. Then, for two independent measurements of ψ by F⊗G, the compound quantum logical entropies can be interpreted as the following “two-measurement” probabilities:

- $h\left(F,G:\psi \right)$ = probability of getting distinct eigenvalues $\phi_{i}\gamma_{j}\neq \phi_{i^{\prime }}\gamma_{j^{\prime }}$ where $i\neq i^{\prime }$ or $j\neq j^{\prime }$;
- $h\left(F|G:\psi \right)$ = probability of getting distinct eigenvalues $\phi_{i}\gamma_{j}\neq \phi_{i^{\prime }}\gamma_{j}$ where $i\neq i^{\prime }$;
- $h\left(G|F:\psi \right)$ = probability of getting distinct eigenvalues $\phi_{i}\gamma_{j}\neq \phi_{i}\gamma_{j^{\prime }}$ where $j\neq j^{\prime }$;
- $m\left(F,G:\psi \right)$ = probability of getting distinct eigenvalues $\phi_{i}\gamma_{j}\neq \phi_{i^{\prime }}\gamma_{j^{\prime }}$ where $i\neq i^{\prime }$ and $j\neq j^{\prime }$.

All the quantum logical entropies have been defined by the general method using the information subspaces, but in the first three cases $h\left(F:\psi \right)$, $h\left(G:\psi \right)$ and $h\left(F,G:\psi \right)$, the density matrix method of defining logical entropies could also be used. Then, the fundamental theorem could be applied relating the quantum logical entropies to the zeroed entities in the density matrices indicating the eigenstates distinguished by the measurements.

The previous set identities for disjoint unions now become subspace identities for direct sums such as:

$$\left[qudit\left(\pi \right)\cup qudit\left(\sigma \right)\right]=\left[qudit\left(\pi \right)-qudit\left(\sigma \right)\right]⊕\left[qudit\left(\pi \right)\cap qudit\left(\sigma \right)\right]⊕\left[qudit\left(\sigma \right)-qudit\left(\pi \right)\right].$$

Hence, the probabilities are additive on those subspaces as shown in Figure A2:

$$h\left(F,G:\psi \right)=h\left(F|G:\psi \right)+m\left(F,G:\psi \right)+h\left(G|F:\psi \right).$$
